# Lipoteichoic acid deficiency permits normal growth but impairs virulence of *Streptococcus pneumoniae*

**DOI:** 10.1038/s41467-017-01720-z

**Published:** 2017-12-12

**Authors:** Nathalie Heß, Franziska Waldow, Thomas P. Kohler, Manfred Rohde, Bernd Kreikemeyer, Alejandro Gómez-Mejia, Torsten Hain, Dominik Schwudke, Waldemar Vollmer, Sven Hammerschmidt, Nicolas Gisch

**Affiliations:** 1 Department of Molecular Genetics and Infection Biology, Interfaculty Institute for Genetics and Functional Genomics, University of Greifswald, Friedrich Ludwig Jahnstr. 15a, 17487 Greifswald, Germany; 2 Division of Bioanalytical Chemistry, Priority Area Infections, Research Center Borstel, Leibniz-Center for Medicine and Biosciences, Parkallee 1-40, 23845 Borstel, Germany; 3 Central Facility for Microscopy, HZI - Helmholtz Centre for Infection Research, Inhoffenstraße 7, 38124 Braunschweig, Germany; 4 University Medicine, Institute of Medical Microbiology, Virology and Hygiene, Rostock University, Schillingallee 70, 18057 Rostock, Germany; 5 Institute for Medical Microbiology, Justus-Liebig University of Giessen, Schubertstraße 81, 35392 Giessen, Germany; 6 Centre for Bacterial Cell Biology, Institute for Cell and Molecular Biosciences, Newcastle University, Richardson Road, Newcastle upon Tyne, NE2 4AX UK

## Abstract

Teichoic acid (TA), a crucial cell wall constituent of the pathobiont *Streptococcus pneumoniae*, is bound to peptidoglycan (wall teichoic acid, WTA) or to membrane glycolipids (lipoteichoic acid, LTA). Both TA polymers share a common precursor synthesis pathway, but differ in the final transfer of the TA chain to either peptidoglycan or a glycolipid. Here, we show that LTA exhibits a different linkage conformation compared to WTA, and identify TacL (previously known as RafX) as a putative lipoteichoic acid ligase required for LTA assembly. Pneumococcal mutants deficient in TacL lack LTA and show attenuated virulence in mouse models of acute pneumonia and systemic infections, although they grow normally in culture. Hence, LTA is important for *S. pneumoniae* to establish systemic infections, and TacL represents a potential target for antimicrobial drug development.

## Abstract (web summary)

Teichoic acid is bound to peptidoglycan (wall teichoic acid, WTA) or to membrane glycolipids (lipoteichoic acid, LTA) in most Gram-positive bacteria. Here, the authors identify a putative ligase required for the assembly of LTA, but not WTA, and important for *Streptococcus pneumoniae* virulence in mouse models.

## Introduction

S*treptococcus pneumoniae* (the pneumococcus) is a human pathobiont that not only colonizes asymptomatically the respiratory tract, but is also capable of causing diseases such as otitis media, acute sinusitis, pneumonia, meningitis, and sepsis^1,2^. The cell surface of this Gram-positive bacterium consists of a peptidoglycan-wall teichoic acid (PGN-WTA) cell wall and is shielded by a capsular polysaccharide (CPS), allowing the cell to evade recognition by the innate immune system of the host^3^. Like most Gram-positive bacteria *S. pneumoniae* also possesses a lipoteichoic acid (LTA), which is anchored to the cell membrane by a glycolipid moiety. Both types of teichoic acid (TA) bind choline-binding proteins (CBPs), an important class of cell surface proteins involved in peptidoglycan remodeling and interactions with host factors. In contrast to many other Gram-positive bacteria, pneumococci contain a structurally unique, complex LTA^4^. Similar LTA structures are known only for other members of the mitis group of streptococci, *Streptococcus mitis* ^5^ and *Streptococcus oralis* ^6^. Pneumococcal LTA (pnLTA) is composed of a lipid anchor (α-D-Glc*p*-(1 → 3)-diacylglycerol) and 4 to 8 repeating units (RUs), each one consisting of a *pseudo*-pentasaccharide ((→4)-6-*O*-*P*-Cho-α-D-Gal*p*NAc-(1 → 3)-6-*O*-*P*-Cho-β-D-Gal*p*NAc-(1 → 1)-Rib-ol-5-*P*-(*O* → 6)-β-D-Glc*p*-(1 → 3)-AATGal*p*-(1 → )). The terminal RU is found both with and without a 6-*O*-phosphorylcholine (6-*O*-*P*-Cho) substitution. The first RU is β-1-linked via the AATGal*p* to the lipid anchor, whereas all other RUs are α-1-linked^7^. Pneumococcal strains containing only one *P*-Cho per RU lack the *P*-Cho at β-D-Gal*p*NAc^8^. Some of the hydroxyl groups of Rib-ol-5-*P* are substituted with D-Ala^7,9^. The RUs of pneumococcal WTA (pnWTA) have the same chemical structure as the RUs of the LTA, but it is not clear whether there is a specific linkage unit between WTA chains and PGN as in other bacteria^10,11^. Based on chemical hydrolysis experiments, it was proposed that pnWTA is linked to the PGN by a phosphodiester to the hydroxyl group at C-6 of the MurNAc^10^, which is thought to be the general mechanism for WTA attachment (reviewed in ref. ^12^). However, cell wall fragments containing an intact linkage between pnWTA and PGN were not isolated or analyzed in previous studies and therefore the nature of this linkage has remained elusive^10,13,14^.

A recent bioinformatic analysis indicates that the pneumococcal TA (pnTA) precursor chains are synthesized by a shared biosynthetic pathway^15^. A key step involves the transport of an undecaprenyl-diphosphate(Und-*PP*)-linked TA precursor across the cytoplasmic membrane by TacF (for “teichoic acid flippase”), which belongs to a family of polysaccharide transmembrane transporters^16^. Members of the LytR-Cps2A-Psr (LCP) protein family have been proposed to attach anionic polymers to PGN in Gram-positive bacteria^17^. The absence of all LCP orthologues from *Staphylococcus aureus* (LytR, CpsA, and Psr) leads to the secretion of WTAs to the extracellular medium, thereby reducing significantly the phosphate content of the cell envelope^18^. All three pneumococcal LCP orthologues appear to have semi-redundant roles in retaining the pneumococcal CPS at the cell surface. It was suggested that the three LCP proteins attach CPS and TA polymers to PGN, and that the LCP enzymes are required to form the LTA^19^. However, another protein, RafX (SPD_1672 in strain D39, SP_1893 in strain TIGR4), has been proposed to assemble pnWTA based on the reduced amount of WTA detected by an antibody in RafX-deficient strains, which also showed impaired colonization capabilities, growth defects, and attenuation in virulence^20,21^.

Here we report the elucidation of the linkage between pnWTA and PGN, which is different in its configuration compared to the linkage between TA chains and the glycolipid anchor in pnLTA. We have also analyzed pnTAs from a nonencapsulated strain and the isogenic *rafX* mutant by high-resolution mass spectrometry (MS) and nuclear magnetic resonance (NMR) spectroscopy. We show that RafX (SPD_1672, SP_1893) is required for the synthesis of pnLTA, but not pnWTA. We propose that this protein is most likely involved in ligation of pnTA precursor chains onto the glycolipid anchor, and rename RafX as TacL (for “lipoteichoic acid ligase”). Furthermore, we show that *tacL* mutants grow with normal rate and morphology in culture but are attenuated in two mouse models of infection.

## Results

### Linkage structure of pnWTA to PGN

**Fig. 1:**
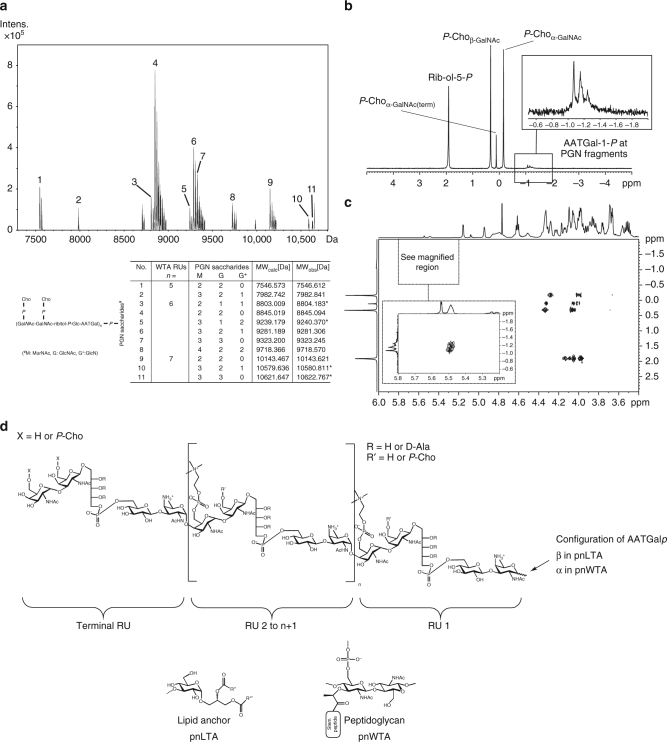
Structural analysis of pnWTA bound to small PGN saccharides from *S. pneumoniae* D39*∆cps∆lgt* and chemical structures of pnTAs. **a** Section of the charge deconvoluted ESI-FT-ICR-MS spectrum (acquired in negative-ion mode). Signals for molecules **1**–**11** represent pnWTA with 5–7 RUs bound to small PGN-derived saccharides; observed and calculated masses are given. *only second isotopic peak was observable. **b** Section (δ_P_ 5.0-(−5.0)) of the ^31^P NMR including assignment of signals. Magnification shows ^31^P NMR signals that are not observable in pnLTA preparations^7^ (compare Fig. 2). **c** Section (δ_H_ 6.00-3.40; δ_P_ 3-(−2)) of the respective ^1^H, ^31^P HMQC NMR spectrum. The important region for the linkage phosphate is magnified. Cross correlation of the phosphates between δ_P_ −1.0 and −1.3 into the broadened signal in ^1^H NMR (δ_H_ 5.51–5.47) could be assigned to the anomeric proton of α-AATGal*p*, proving the stereochemistry of this bridging phosphate is an α-1-linked atom. **d** Chemical structures of pnTAs. RUs of both TAs contain the same pseudo-pentasaccharide building blocks, whereby the terminal RU can occur with or without 6-*O*-*P*-Cho-substitution on both Gal*p*NAc. A few mono-*P*-Cho-substituted TAs lack the *P*-Cho at β-Gal*p*NAc (R′). In pnLTA, the first RU is β-1-linked to the lipid anchor (α-D-Glc*p*-(1 → 3)-DAG); consistent with the α-configuration of the respective linkage of pnWTA to PGN. All other RUs are α-glycosidically linked to the previous one. Hydroxyl groups of ribitol can be substituted by D-alanine (D-Ala; R). n number of RU; R′′ and R′′′, alkyl or alkenyl residues of fatty acid chains in lipid anchor (summarized in ref. ^8^)

In order to determine the linkage structure of pnWTA, we isolated the PGN-WTA complex of *S. pneumoniae* D39Δ*cps*Δ*lgt* using a previously published procedure^11^. This strain lacks the CPS and the gene encoding for the lipoprotein diacylglyceryl transferase (Lgt) and is therefore deficient in lipidation of prelipoproteins^7,22^. Isolated pnLTA from this strain was shown to be structurally identical with that of its parental strain D39Δ*cps* and to be free of Toll-like receptor 2 stimulating activity^7^. Therefore, we considered this strain to be best suitable for the investigation of the PGN-WTA complex and for prospective cell stimulation assays, avoiding possible contamination with lipoproteins. The cell wall was digested with pneumococcal amidase LytA and the resultant peptide-free PGN glycan chains carrying pnWTA were isolated by gel permeation chromatography (GPC) (Supplementary Fig. 1a). This material was digested with lysozyme and mutanolysin, producing pnWTA chains bound to a variety of small PGN fragments. The mixture was further purified by another GPC step (Supplementary Fig. 1b). Figure 1a shows the relevant section of the mass spectrum obtained from this material. The identified molecules correspond to pnWTA chains with five to seven RUs bound by a phosphate moiety to di-, tri -, or tetramers of MurNAc-GlcNAc disaccharides.

Analysis by ^31^P NMR revealed the occurrence of additional signals when compared to de-*O*-acylated pnLTA isolated from the same strain^7^ in the chemical shift range between δ_P_ −1.0 and −1.3 (Fig. 1b). Using a ^1^H, ^31^P-correlated heteronuclear multiple quantum correlation (HMQC) NMR experiment (Fig. 1c), this bridging phosphate was determined to be an α-1-phosphate because of the cross correlation into the broadened signals (caused by the heterogeneity of the PGN sugar fragments) in the ^1^H NMR spectrum between δ_H_ 5.51 and 5.47 ppm. Based on further NMR analysis, we assigned this α-1-phosphate to AATGal*p*, which verifies the direct linkage of pnWTA to PGN. The respective ^1^H, ^13^C HSQC NMR spectrum is shown in Supplementary Fig. 2; the complete NMR chemical shift data for the pnWTA are listed in Supplementary Table 1. The structural elucidation of this linkage clarified the last remaining question about the chemical structure of pnTAs: in the final model (Fig. 1d), both TA types contain the same pseudo-pentasaccharide building blocks, whereas the terminal RU can occur with or without 6-*O*-*P*-Cho-substitution on both Gal*p*NAc moieties. In pnLTA, the first repeating unit is β-1-linked to the lipid anchor (α-D-Glc*p*-(1 → 3)-DAG). By contrast, the pnWTA is linked directly—without the presence of an additional linkage unit—to the PGN by an AATGal*p*-α-1-phosphate. These different anomeric linkages (β-1 and α-1, respectively) for pnLTA and pnWTA implies that different ligases probably catalyze the linkage reactions for the two pnTAs.

### *TacL* mutants lack pnLTA and attach pnWTA to PGN

Based on the different linkage structures of pnWTA and pnLTA, we hypothesized that the ligase for pnLTA assembly on the glycolipid is not an LCP phosphotransferase enzyme. A candidate gene encoding the LTA ligase was recently identified as *rafX* ^20^, hereafter called *tacL*. Hence, we isolated pnLTA from the isogenic *tacL* mutants D39Δ*cps*Δ*tacL* and TIGR4Δ*cps*Δ*tacL*, their parental strains and the complemented mutants (Supplementary Table 2) as described^7^. The *tacL* deletion and its complementation were verified in the respective encapsulated and non-encapsulated *S. pneumoniae* D39 strains by real-time quantitative PCR (qRT-PCR; Supplementary Fig. 3).

**Fig. 2:**
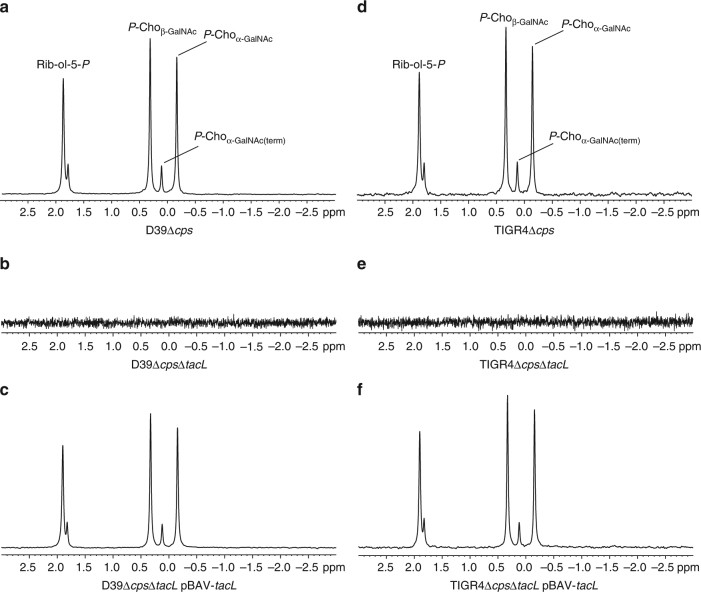
^31^P NMR spectra of pnLTA preparations. Sections (δ_P_ 3-(−3)) of ^31^P NMR spectra measured from LTA isolated from pneumococcal strains **a** D39Δ*cps*, **b** D39Δ*cps*Δ*tacL*, **c** D39Δ*cps*Δ*tacL* pBAV-*tacL*, **d** TIGR4Δ*cps*, **e** TIGR4Δ*cps*Δ*tacL*, and **f** TIGR4Δ*cps*Δ*tacL* pBAV-*tacL*. **a**, **c**, **d**, **f** Hydrazine-treated LTA was used, for *tacL* knockout mutants (**b**, **e**) the typical LTA-containing fractions from the HIC purifications (compare Supplementary Fig. 4) were measured without prior hydrazine treatment

The chromatogram in Supplementary Fig. 4 is a representative hydrophobic interaction chromatography (HIC) purification of pnLTA from strain D39Δ*cps*; the normalized phosphate content is depicted in the upper panel. The ^31^P NMR spectra of the corresponding hydrazine-treated pnLTAs are shown in Fig. 2. The *tacL* mutants lacked detectable amounts of pnLTA (Fig. 2b, e), while pnLTA was present in both the complemented (Fig. 2c, f) and the isogenic parental strains (Fig. 2a, d) indicating that TacL is required for LTA synthesis. The MS analysis of hydrazine-treated pnLTA from in trans-complemented and parental strains resulted in nearly identical profiles indicating that the expressed *tacL* gene was functional (Supplementary Fig. 5). Moreover, the ^31^P NMR spectra of the PGN-WTA complex after LytA digestion (Supplementary Fig. 6) prove that pnWTA was present in all investigated strains, including the *tacL* mutants, thus showing that TacL is not needed for the synthesis of the WTA-PGN linkage.

The genomes of the nonencapsulated *tacL* mutants and their isogenic parental strains were sequenced and compared to exclude the possibility of compensatory mutations. For strain D39Δ*cps*Δ*tacL*, we identified four single-nucleotide polymorphisms (SNPs) in comparison to its parental strain D39Δ*cps* (Supplementary Table 3). Two are located in intergenic regions, the two others are placed in *spd_0768* (Asp297Gly) and *spd_1179* (Ala141Thr), respectively. The latter encodes a predicted lanthionine synthetase, whereas Spd_0768 (CozE) has recently been described as a member of the MreCD complex of *S. pneumoniae* that directs the activity of PBP1a to the mid-cell plane, where it promotes zonal cell elongation and normal morphology^23^. The identified variants in the genome of strain TIGR4Δ*cps*Δ*tacL* are listed in Supplementary Table 4. Beside three variations in intergenic regions, our analysis identified three SNPs in *sp_1894* and two SNPs in the pseudogene *sp_rs12410* directly located in between *tacL* and *sp_1894*, with a small overlap with the end of the ORF of *sp_1894*. However, PCR amplification and DNA sequencing of these specific regions disproved two of these SNPs. The confirmed SNPs in *sp_1894*, encoding GtfA (a transferase involved in glycosylation of pneumococcal serine-rich repeat adhesins with *O*-linked *N*-acetyl-D-glucosamine^24^), are Ile370Val and a silent one (Glu432Glu). Furthermore, a 1 bp deletion causing a frameshift in *sp_1914*, encoding a putative membrane protein of unknown function, was identified. None of these mutations are likely to cause any alteration in the pnTA structure.

In summary, whole-genome sequencing and subsequent analysis of genomic variations in the *tacL* mutants apparently excluded secondary mutations with expectable impact on pnTA biosynthesis, especially because none of the observed variations and therefore potentially compensatory mutations took place in both serotypes. Taken together, our data support the hypothesis that TacL is involved in pnLTA assembly, and not in pnWTA assembly as was previously proposed^20^.

### Loss of TacL does not perturb pneumococcal physiology

The loss of LTA is lethal to *S. aureus* under standard laboratory conditions^25^. Hence, we tested the impact of *tacL* deletion on pneumococcal growth in two complex media and a chemically defined medium (RPMI_modi_)^26^ (Supplementary Fig. 7a). TacL deficiency slightly affected growth of the encapsulated D39 *tacL* mutant in RPMI_modi_. However, this effect could not be observed for the nonencapsulated strain or for the growth of the encapsulated and nonencapsulated strains in the two complex media. The complemented, nonencapsulated mutant revealed reduced growth in RPMI_modi_ compared to both the wild-type and the TacL-deficient strain. This is possibly an effect of the plasmid-based in trans complementation of the mutant due to a gene dosage effect of *tacL* by multiple plasmid copies. The *tacL* mutant was slightly affected in detergent-induced autolysis, increasing the rate of cell lysis in the presence of 0.01% Triton X-100 in the encapsulated strains in comparison to the wild-type strain. In the nonencapsulated strains, the effect was less pronounced (Supplementary Fig. 7b).

**Fig. 3:**
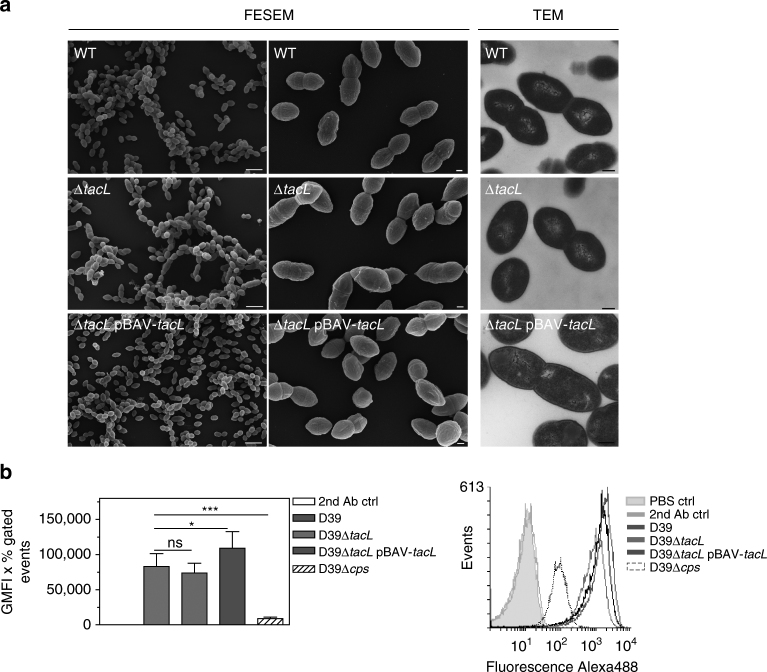
Analysis of pneumococcal cell morphology and cell division by electron microscopy and quantification of the capsule content. **a** FESEM and TEM ultrathin sections revealed no visible differences in the cell morphology and cell division septa morphology between nonencapsulated D39 wild-type (WT), *tacL* mutant (Δ*tacL*), or complemented mutant (Δ*tacL* pBAV-*tacL*) grown in THY medium. Scale bars = 2 µm (FESEM, left panel), 0.2 µm (FESEM, right panel), 0.2 µm (TEM). **b** Flow cytometric analysis of the capsule content. Bacteria were grown in THY medium to *A*_600_ = 0.35–0.45, washed and incubated with serum against type 2 capsule followed by a secondary Alexa_488_-labeled antibody. The values are represented as the geometrical mean fluorescence intensity multiplied with percent-gated events. Results are expressed as means ± s.d. (each experiment was performed at least three times). ***p < 0.001; *p < 0.05; ns not significant (Student's unpaired *t* test)

We next analyzed by field emission scanning and transmission electron microscopy (FESEM, TEM) how LTA deficiency affects pneumococcal cell morphology and cell division in strain D39Δ*cps* grown in complex medium (Fig. 3a) or in RPMI_modi_ (Supplementary Fig. 8). No significant differences in cell size or the localization of division septa between the parental strain, the *tacL* mutant, and complemented mutant were observed for both growth conditions. Furthermore, the amount of capsule was similar in strains with or without *tacL*, as determined by a flow cytometric assay with an antiserum directed against the capsule type 2 (Fig. 3b).

**Fig. 4:**
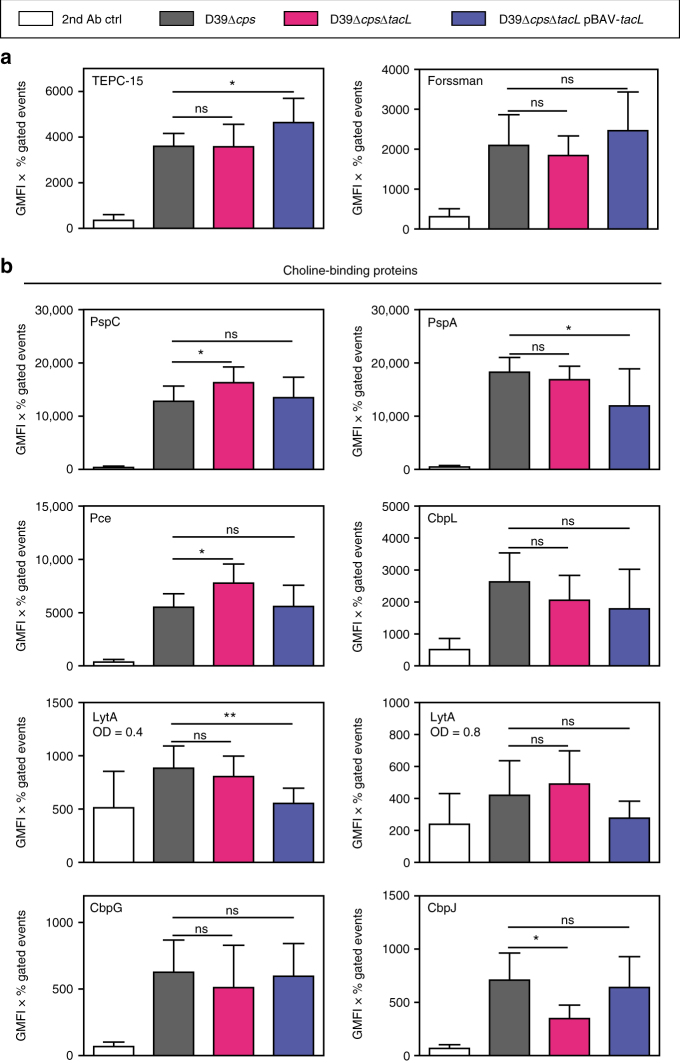
Quantitative analysis of teichoic acids and choline-binding proteins. Bacteria were grown in THY medium to *A*_600_ = 0.35–0.45 (for LytA 0.4 and 0.8), washed and incubated with antibodies. **a** The amount of *P*-Cho and the Forssman antigen of teichoic acids were determined using specific primary antibodies (TEPC-15, anti-Forssman) and secondary Alexa_488_-labeled antibody in a flow cytometry-based assay. **b** Surface-associated CBPs were analyzed using specific polyclonal mice IgG against the individual CBPs and secondary Alexa_488_-labeled antibodies in a flow cytometry-based assay (see also Supplementary Fig. 9 for representative histograms). The values are represented as the geometrical mean fluorescence intensity multiplied with percent-gated events. Results are expressed as means ± s.d. (each experiment was performed at least three times). *p < 0.05; ns not significant (Student's unpaired *t* test)

It has been proposed that WTA represent ~90% of the total TA content in *S. pneumoniae* ^27^; a similar WTA/LTA ratio was determined for its close relative *S. oralis* ^28^. If LTA constitutes about 10% of pneumococcal TAs, the complete lack of LTA in the *tacL* mutant may not lead to a significant decrease in the measurable *P*-Cho content of the pneumococcal cell wall. We quantified the amount of *P*-Cho in the cell wall of the different strains with the *P*-Cho-specific antibody TEPC-15 (Fig. 4a and Supplementary Fig. 9a). All strains contained a similar amount of *P*-Cho. Unfortunately, pnTA-specific antibodies are not currently available. For further quantification of the TAs, we made use of an anti-Forssman antibody that recognizes the terminal sugar residues (α-D-Gal*p*NAc-(1 → 3)-β-D-Gal*p*NAc-(1 → )) of pnWTA and pnLTA^7^. Comparable amounts of the Forssman antigen were detected by flow cytometry in the *tacL* mutant, wild-type and complemented mutant. In summary, the loss of LTA in the *tacL* mutant appears to be compensated either by a higher abundance of WTA on the cell surface or by the accumulation of TA precursor chains. Another possibility could be that the loss of pnLTA is simply not measureable due to its low abundance in comparison to the pnWTA. At any rate, we can exclude a dramatic decrease of the cell wall *P*-Cho content in *tacL* mutants.

The amount of cell surface-attached CBPs depends on the amount of *P*-Cho-loaded TAs, which serve as an anchor for these proteins. We determined the presence and amount of various CBPs on the cell surface of the nonencapsulated strains using specific polyclonal antibodies against different CBPs (Fig. 4b and Supplementary Figs. 9b and 10). The pneumococcal surface proteins PspC and Pce were slightly, but not significantly, enhanced in the *tacL* mutant, whereas CbpJ (a protein involved in host–pathogen interaction)^29^ was slightly reduced. Several other CBPs such as PspA, the major autolysin LytA (at *A*_600_ 0.4 and 0.8) and the CBPs CbpL and CbpG were unaltered in the *tacL* mutant, consistent with the similar content of *P*-Cho and TA chain ends as determined with the antibodies. Taken together, these results suggest that the *tacL* mutant carries largely unaltered amounts of *P*-Cho in its cell wall despite the absence of LTA.

### Influence of pnLTA on pneumococcal adhesion and phagocytosis

To elucidate the role of pnLTA in bacterial adhesion to human lung epithelial cells, A549 cells were infected with the nonencapsulated D39 strain, its isogenic *tacL* mutant, or its complemented mutant. Immunofluorescence microscopy revealed that the TacL-deficient strain showed a significant decrease in the ability to adhere to A549 cells after 2 or 4 h of infection, whereas the adherence of the complemented mutant was comparable to that of the parental strain (Supplementary Fig. 11).

The impact of LTA on pneumococcal phagocytosis by PMA-differentiated (phorbol 12-myristate 13-acetate) THP-1 cells was evaluated in antibiotic protection assays and by double immunofluorescence staining. A time-dependent uptake of pneumococci could be monitored for all tested strains with a moderate increase for the complemented mutant in the antibiotic protection assay compared to the parental strain D39Δ*cps* (Supplementary Figs. 12a and 13). The intracellular killing of the TacL-deficient mutant within the phagocytes was moderately but significantly decreased after 1 or 2 additional hours of incubation as determined by antibiotic protection assays (Supplementary Fig. 12b). In conclusion, pneumococcal adherence to epithelial cells is significantly affected by the loss of LTA, whereas bacterial uptake by phagocytes was not affected.

### Loss of TacL attenuates pneumococcal virulence

**Fig. 5:**
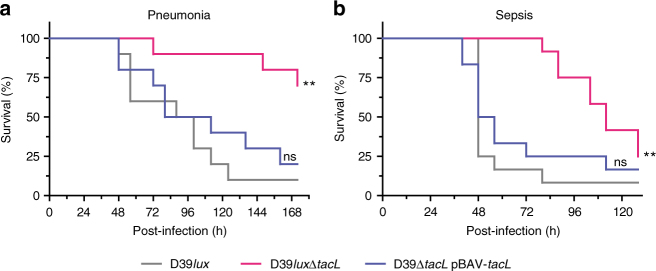
Impact of TacL deficiency on pneumococcal virulence. Survival of CD-1 mice after **a** intranasal infection with ~2.5 × 10^7^ bioluminescent *S. pneumoniae* D39*lux* wild type, D39*lux*Δ*tacL*, or the complemented mutant (D39Δ*tacL* pBAV-*tacL*) in the pneumonia model (*n* = 10) (see also Supplementary Fig. 14 for bioluminescence images). **b** Survival of CD-1 mice (*n* = 12) after intraperitoneal infection with ~3 × 10^3^ pneumococci in the systemic infection model. **p < 0.01; ns not significant (Log-rank (Mantel–Cox) test)

We used two different mouse models of infection to assess the impact of the TacL deficiency on pneumococcal pathogenesis (Fig. 5a, b and Supplementary Fig. 14). In the acute pneumonia model (Fig. 5a, Supplementary Fig. 14), mice were infected intranasally with *S. pneumoniae* D39*lux*, D39*lux*Δ*tacL*, or the complemented mutant. In case of the wild type, 90% of the infected mice developed pneumonia, which resulted in severe sepsis and death of the mice. By contrast, only 30% of the mice infected with the *tacL* mutant showed severe signs of pneumonia and sepsis. The course of disease is clearly prolonged in mice infected with the TacL-deficient strain. Importantly, complementing the *tacL* mutant in trans restored the phenotype of the wild type. In the systemic infection (sepsis) model (Fig. 5b), mice were infected intraperitoneally with the encapsulated D39*lux* strain, D39*lux*Δ*tacL*, or the complemented mutant and the severity of disease was monitored over time. Mice infected with the wild-type strain or the complemented mutant showed earlier onset of disease and died significantly sooner than mice infected with the *tacL* mutant. In summary, loss of TacL resulted in a significantly attenuated virulence, indicating an essential role of LTA in the pathophysiology of pneumococci.

## Discussion

**Fig. 6:**
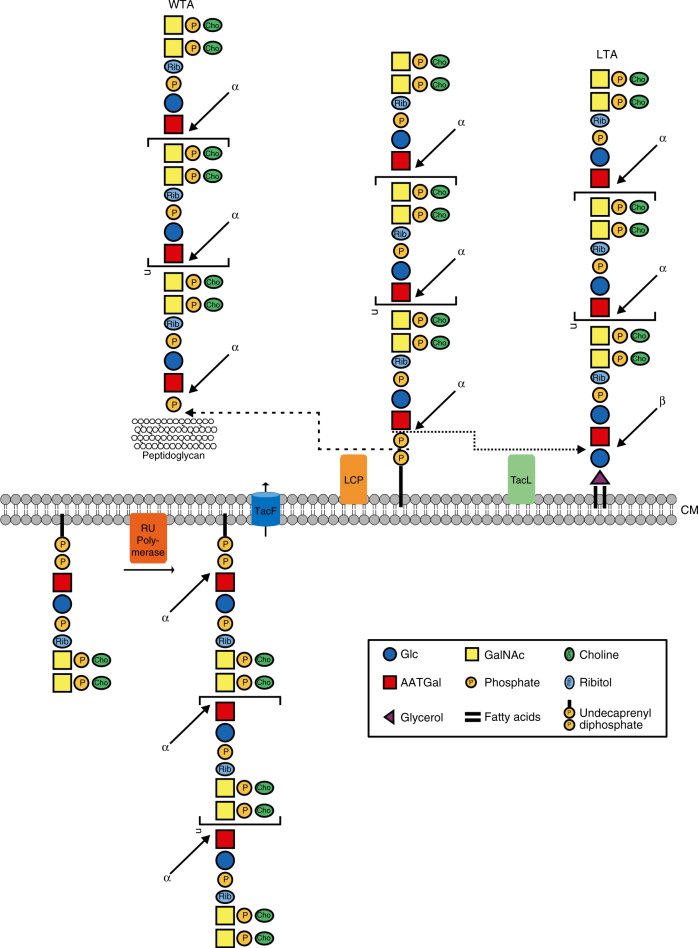
Last steps in the TA biosynthesis pathway in *S. pneumoniae*. The Und-*PP*-linked monomeric repeats are polymerized by the RU polymerase and transported through the cytosolic membrane by TacF. The complete biosynthesis pathway is reviewed and discussed in ref. ^15^. Finally, the pnTA precursor chain is transferred onto the PGN to form the pnWTA or onto the glycolipid anchor to form the pnLTA, respectively. The transfer to the PGN is performed—most likely in a semi-redundant manner^19^—by the LCP proteins Psr, LytR, and Cps2A. Here the TA chain is transferred including one phosphate, thus retaining the anomeric α-configuration of the AATGal*p* moiety of the first RU. In pnLTA formation, a new glycosidic bond between the glucose moiety of the glycolipid anchor and the first AATGal*p* is formed. We propose that TacL may catalyze this reaction, which inverts the stereochemistry of the anomeric carbon leading to the β-configuration of the AATGal*p* moiety of the first RU in pnLTA. From these observations, it can be concluded that the Und-*PP*-linked pnTA precursor chains are synthesized with all AATGal*p* moieties in the α-configuration. AATGal 2-acetamido-4-amino-2,4,6-trideoxygalactose, CM cytoplasmic membrane, GalNAc *N*-acetylgalactosamine, Glc glucose, LCP LytR-Cps2A-Psr family protein

We show in this work that TacL (also known as RafX, SPD_1672 (strain D39) or SP_1893 (in TIGR4)) is required for pnLTA formation in *S. pneumoniae*. We propose that TacL is a lipoteichoic acid ligase acting in the final step of pnLTA synthesis (Fig. 6). Our data are not consistent with the previously hypothesized role of SPD_1672 and its homologs in the biosynthesis of pnWTA, which was based on antibody detection methods and did not involve a detailed structural analysis of WTA or LTA. Remarkably, with the exception of few choline-utilization enzymes^30^, most of the enzymes in the predicted pathway of pnTA synthesis^15^ remain uncharacterized.

The undecaprenyl-diphosphate (Und-*PP*)-linked monomeric repeats are polymerized by the RU polymerase, transported through the cytosolic membrane by TacF and are finally transferred onto the PGN or the glycolipid anchor. We show here that the linkage conformation in pnWTA differs from that of pnLTA; the AATGal*p* moiety within the first RU of pnLTA is β-configurated^7^, while the respective AATGal*p* in pnWTA has the α-configuration. This observation suggests that the final reactions in pnLTA and pnWTA assembly differ mechanistically and that the reactions are likely catalyzed by enzymes from different classes. The transfer of the TA precursor chains onto the PGN is most likely performed by the LCP proteins Psr, LytR, and Cps2A^19^, which belong to the same family of phosphotransferases. Here the TA chain is transferred with one phosphate, thus retaining the anomeric configuration of the AATGal*p* moiety of the first RU. By contrast, in LTA synthesis, a new glycosidic bond is formed between the glucose moiety of the glycolipid anchor and the first AATGal*p*. This reaction inverts the stereochemistry of the anomeric carbon, leading to the β-configuration of the AATGal*p* moiety of the first RU in pnLTA. A similar mechanism has been described in the biosynthesis of the lipopolysaccharide (LPS) of Gram-negative bacteria when the O-antigen chain is attached to the core oligosaccharide. The O-antigen is synthesized as an Und-*PP*-linked precursor and is translocated across the cytosolic membrane by different pathways^31^. In several published LPS structures, the first sugar of the O-antigen always has the β-configuration, whereas it has the α-configuration in the Und-*PP*-linked precursor^32^. This reaction is catalyzed by the O-antigen ligase WaaL (RfaL).

TacL of *S. pneumoniae* may be located in the cytoplasmic membrane with nine transmembrane helices (as predicted by TMbase25; http://embnet.vital-it.ch/software/TMPRED_form.html)^33^ and a Wzy_C Pfam domain (STRING database v. 10.0). Wzy_C superfamily domains are characteristic of enzymes involved in the synthesis of O-antigens in Gram-negative bacteria, including O-antigen ligases. Pneumococcal TacL is composed of 397 amino acids, comparable to the length of the RfaL O-antigen ligase of *E. coli* K12 (419 aa) or WaaL of *Pseudomonas aeruginosa* PAO1 (401 aa), which are predicted by TMbase to have similar secondary structures to TacL. TacL shares significant sequence similarities to a stretch of 208 amino acids in RfaL (21.6% identity, 57.7% similarity) and to a 378 amino acid stretch in WaaL (18.8% identity, 49.2% similarity). The attachment of carbohydrate polymers to glycolipids to form complex cell wall glycopolymers in Gram-positive and Gram-negative bacteria thus appears to share a conserved mechanism.

The repeating units of the wall and lipoteichoic acids of pneumococci comprise identical RUs, which is a special feature that differs from most other Gram-positive bacteria. Most of the Firmicutes, like *S. aureus*, possess WTAs built up of ribitol-phosphate RUs, whereas the LTA comprises repeating glycerol–phosphate units^34,35^. The total loss of WTAs in *S. aureus* leads to reduced adherence to host cells, while LTA is essential for growth and cell division^25,36,37^. Here we show that a *S. pneumoniae tacL* mutant is unable to anchor the LTA to the membrane glycolipid. Strikingly, all strains showed a comparable growth in complex media, and even in chemically defined medium (RPMI_modi_) only minor differences could be observed. Only the encapsulated TacL-deficient D39 mutant was slightly affected in growth in RPMI_modi_. This observation is in accordance with previous findings from Wu et al.^20^, showing also a slightly delayed growth of a RafX (Spd_1672)-deficient D39 strain in semisynthetic casein hydrolysate medium supplemented with yeast extract. Inconsistent with our study, Wu et al.^20^ described altered cell shape and division for their mutant strain. However, the published *rafX* mutant was not analyzed by whole-genome sequencing, and the authors did not discuss possible secondary mutations affecting morphology^20^. Additionally, the complemented, nonencapsulated strain (D39Δ*cps*Δ*tacL* pBAV-*tacL*) was affected during growth in RPMI_modi_. However, this could be due to the presence of multiple *tacL* gene copies within the complemented mutant. As mentioned, *tacL* mutants generated by us did not exhibit changes in cell morphology or division compared to parental strains, which is different to LTA-deficient *S. aureus* mutants^37^. Hence, contrary to findings in other Gram-positive species, we show here that LTA is not essential in *S. pneumoniae*, which might be due to the structural identity of pnLTA and pnWTA. LTA forms ~10% of total teichoic acid in the pneumococcal cell wall^27^. The total amount of pnTA in the *tacL* mutant was unaffected according to the quantification of *P*-Cho and chain ends using specific antibodies, which suggests that an increase in pnWTA content or the presence of carrier lipid-bound pnTA chains may compensate for the loss of pnLTA. Alternatively, the amount of pnLTA may be too low to be substantiated reliably using currently available methodologies.

Pneumococcal TAs carry *P*-Cho modifications, which are essential for bacterial growth^38^. CBPs represent a group of pneumococcal surface proteins that are non-covalently bound to the *P*-Cho moieties of pnLTA and pnWTA^39^. Several CBPs have important functions in bacterial physiology and virulence^40–42^. Consistent with the quantification of pnTA content, we found that five out of seven tested CBPs were unchanged in abundance in the *tacL* mutant. The amounts of CbpJ, a protein interacting with the C-reactive protein of humans^29^, and the phosphorylcholine esterase Pce were only slightly altered in the *tacL* mutant. Thus, changes in abundance of CBPs can be excluded from the cause of the attenuation of the *tacL* mutant in pneumonia and sepsis infection models. However, the loss of pnLTA may influence the membrane integrity and/or fluidity, which affects survival of mutants deficient in TacL in vivo but cannot be visualized by electron microscopy. In a previous study^7^, we have shown that LTA preparations of a pneumococcal Δ*lgt* mutant had residual immunostimulatory properties when tested in human mononuclear cells (hMNCs), in the absence of TLR2-stimulating activity. This was consistent with an observed stimulation of hMNCs by a synthetic short-chain analog of pnLTA^43^. The receptor(s) and mechanisms of general LTA sensing by the innate immune system have not been investigated in depth. However, some studies suggest the involvement of different pathways of the complement system in the interaction with *S. pneumoniae* and pnTAs (summarized in ref. ^8^).

Potentially, the lack of such interactions with TacL deficient and therefore LTA lacking pneumococcal mutants could contribute to the observed impaired virulence in the mouse sepsis model. The significant decrease in adhesion to human epithelial cells observed in cell culture infections with A549 cells could explain the observed pathophenotype in the pneumonia model. Due to its fundamental and essential function under in vivo conditions, TacL represents a potential target for antimicrobial substances.

## Methods

### Bacterial strains and growth conditions

Bacterial strains are listed in Supplementary Table 2. *Escherichia coli* were cultured on Luria Broth (LB) plates or in liquid LB medium, supplemented with 200 µg ml^−1^ erythromycin at 37 °C. Transformation of *E. coli* with plasmid DNA was carried out using chemically competent cells. *S. pneumoniae* serotype 2 D39 and serotype 4 TIGR4 and their isogenic mutants were grown on Columbia blood agar plates (Oxoid) containing erythromycin (5 µg ml^−1^) and/or chloramphenicol (5 µg ml^−1^), or cultured in Todd-Hewitt broth supplemented with 0.5% yeast extract (THY; Roth) or chemically defined medium (RPMI_modi_^26^; GE Healthcare, Bio-Sciences), respectively. Cultivation of pneumococci on blood agar or in liquid cultures was performed at 37 °C and 5% CO_2_ without agitation.

### Mutant construction

For the construction of the pneumococcal *tacL* mutants in D39 and TIGR4, a DNA fragment consisting of the *S. pneumoniae* D39 *spd_1672* gene and its up- and down-stream flanking regions were amplified by PCR from genomic DNA using primer SPD1672_OLup_for and SPD1672_OLdwn_rev (primers are listed in Supplementary Table 2). The purified PCR products were cloned into pUC18 and *E. coli* DH5α chemically competent cells were transformed with the resulting plasmid. The recombinant plasmid pNH1 harboring the desired DNA insert was purified and used as template for an inverse PCR reaction with primer InvrevKpnISPD1672 and InvforPstISPD1672. The deleted gene sequence was replaced by an *ermB* gene, amplified by PCR from vector pTP1 using primer InvrevKpnIErm and InforPstIErm. The final recombinant plasmid was used to transform and mutagenize pneumococci. Transformation efficiency was evaluated using the final recombinant plasmid vs. another gene deletion plasmid (for deletion of the *cbpL* gene) in *S. pneumoniae* D39Δ*cps* ^44^. Remarkably, the transformation efficiency was thereby significantly increased for the *tacL* deletion (2.8 colonies per ng plasmid for *cbpL* vs. 29.8 colonies per ng plasmid for *tacL*).

Isogenic mutants were complemented by a pBAV1CpE-based in trans system; pBAV1CpE was modified from pBAV1K-T5-gfp^45^, by exchanging the kanamycin-resistance gene for a chloramphenicol-resistance gene and the T5 promotor for an erythromycin promoter region (pE), which includes the ribosomal-binding site and start codon. The complete *spd_1672* gene was amplified by PCR using primer 1672_com_for and Spd1672_com_rev and the purified fragment was cloned into pBAV1CpE. The resulting plasmid pBAV-*tacL* was used to transform isogenic *tacL* mutants. The deletion of *tacL* as well as the in trans complementation were verified using qRT-PCR (Supplementary Fig. 3).

### Real-time quantitative PCR (qRT-PCR)

Encapsulated and nonencapsulated D39 wild-type strain, *tacL*-deficient mutant, and complemented mutant were cultivated in THY until mid-log phase (*A*_600_ = 0.35–0.45) and harvested for RNA isolation using EURx GeneMatrix Universal RNA purification kit (roboklon). The quality of RNA was checked by agarose gel electrophoresis and standard PCR using primer EnoRT_F and EnoRT_R (see Supplementary Table 2). The synthesis of cDNA was performed using the SuperScript III Reverse Transcriptase (ThermoFisher) and hexameric_random primers (GE Healthcare) according to the manufacturer's instructions. The quality of cDNA was controlled by PCR using primer EnoRT_F and EnoRT_R and the concentration was measured by nanodrop. cDNA was stored at −20 °C until further tests. For the qRT-PCR experiments, StepOnePlus^TM^ Real-Time PCR System (Applied Biosystems) and SYBR^®^ Green Master Mix (Biorad) were used in combination with *tacL*-specific primers as well as *enolase*-primers as control (see Supplementary Table 2). The StepOne software (v. 2.3, Life Technologies) was used for data analysis. The final results are presented as magnitude of fluorescence (ΔRn) plotted against PCR cycle numbers.

### Sequencing and bioinformatics analysis

*Sequencing:* 1 ng of purified chromosomal DNA from *S. pneumoniae* strains D39Δ*cps* (PN111), D39Δ*cps*Δ*tacL* (PN601), D39Δ*cps*Δ*tacL* pBAV-*tacL* (PN634), TIGR4Δ*cps* (PN259), TIGR4Δ*cps*Δ*tacL* (PN603), and TIGR4Δ*cps*Δ*tacL* pBAV-*tacL* (PN636) was used to prepare individual libraries employing and following the Illumina Nextera^©^ XT DNA Library Prep Kit. An Agilent Technology 2100 Bioanalyzer served to verify tagmentation and the final library fragment size distribution on a high sensitivity DNA chip. AMPure XP beads were used for DNA library purification. The final pooled library was applied to a MiSeq Reagent v3 600cycle kit and sequenced on a MiSeq system as 300 cycle paired-end run. The final library pool was spiked with 5% PhiX control library. A cluster density of 847 ± 25 (K mm^−2^) was achieved with 96.46 ± 1.48% of clusters passing filter specifications. 20.3 Mio reads (94.7%) of 21.1 Mio total reads passed filter specifications, leading to 12.52 Gbp of sequence data. Index reads were evenly distributed across the six individual samples. Generated FASTQ files were subjected to further bioinformatics analysis as outlined below.

*SNP detection and annotation:* SNP detection was done for *S. pneumoniae* D39Δ*cps*, D39Δ*cps*Δ*tacL*, TIGR4Δ*cps*, TIGR4Δ*cps*Δ*tacL* individually using “snippy” (https://github.com/tseemann/snippy; parameter: minimum portion for variant evidence: 60%; minimum coverage of variant site: ≥5 sequences reads). As reference genome *Streptococcus pneumoniae* D39 (NC_008533.1) or *Streptococcus pneumoniae* TIGR4 (NC_003028.3) was used.

The resulting SNPs for each group of mutants (D39Δ*cps* vs. D39Δ*cps*Δ*tacL*) and (TIGR4Δ*cps* vs. TIGR4Δ*cps*Δ*tacL*) were merged and compared. Genome coverage was estimated with qualimap^46^ using the same reference genomes as for SNP detection.

### Isolation of pneumococcal teichoic acids

*Extraction and Isolation of LTA:* LTA purification was performed basically as described elsewhere^7^, but to optimize yield of pnLTA, one specific detail has been modified. Pneumococcal cells were resuspended in citrate buffer (50 mM, pH 4.7) and disrupted three times by French press (Constant Cell Disruption System, Serial No. 1020) at 10 °C at a pressure of 20 kPSI. SDS was added to a final concentration of 4% to the combined supernatants. The solution was incubated for 30 min at 100 °C and was stirred afterwards overnight at room temperature. The solution was centrifuged at 30,000×*g* for 15 min at 4 °C. The pellet was washed four times with citrate buffer using the centrifugation conditions as above. The combined LTA-containing supernatants and the resulting sediment, containing the crude PGN-WTA complex, were lyophilized separately. The resulting solids were both washed five times with ethanol (centrifugation: 20 min, 20 °C, 10,650×*g*) to remove SDS and lyophilized (leading to pellet A containing LTA and pellet B containing the PGN-WTA complex). For LTA isolation, pellet A was resuspended in citrate buffer and extracted with an equal volume of butan-1-ol (Merck) at room temperature under vigorous stirring. The phases were separated by centrifugation at 2,100×*g* for 15 min at 4 °C. The aqueous phase (containing LTA) was collected, and the extraction procedure was repeated twice with the organic phase plus interphase. The combined aqueous phases were lyophilized and subsequently dialyzed for 5 days at 4 °C against 50 mM ammonium acetate buffer (pH 4.7; 3.5 kDa cutoff membrane); the buffer was changed every 24 h. The resulting crude LTA was purified further by hydrophobic interaction chromatography (HIC) performed on a HiPrep Octyl-Sepharose column (GE Healthcare; 16 × 100 mm, bed volume 20 ml). The crude LTA material was dissolved in as little starting buffer (15% propan-1-ol (Roth) in 0.1 M ammonium acetate (pH 4.7)) as possible and centrifuged at 13,000×*g* for 5 min at room temperature and the resulting supernatant was lyophilized. The LTA-containing pellet was dissolved in the HIC starting buffer at a concentration of 30 mg ml^−1^ and purified by HIC using a linear gradient from 15 to 60% propan-1-ol (Roth) in 0.1 M ammonium acetate (pH 4.7). LTA-containing fractions were identified by a photometric phosphate test^47^. The phosphate-containing fractions were combined, lyophilized, and washed with water upon freeze drying to remove residual buffer.

*Extraction and isolation of WTA:* WTA isolation and extraction was carried out as described elsewhere^11^, but with minor modifications. Pellet B (containing the crude PGN-WTA complex), which arose during LTA isolation, was resuspended at a concentration of 10 mg ml^−1^ in 100 mM Tris-HCl (pH 7.5) containing 20 mM MgSO_4_. DNase A and RNase I were added to final concentrations of 10 and 50 µg ml^−1^, respectively. The suspension was stirred for 2 h at 37 °C. Subsequently, 10 mM CaCl_2_ and trypsin (100 µg ml^−1^) were added and the stirring was continued overnight at 37 °C. SDS at a final concentration of 1% was added, and the mixture was incubated for 15 min at 80 °C to inactivate the enzymes. The cell wall was recovered by centrifugation for 45 min at 130,000×*g* at 25 °C. The resulting pellet was resuspended in 0.8 ml 8 M LiCl per 1 ml initially used Tris-HCl solution and incubated for 15 min at 37 °C. After another centrifugation using the same conditions as above, the pellet was resuspended in 1 ml 10 mM ethylenediaminetetraacetic acid (EDTA, pH 7.0) per ml of the Tris-HCl solution used initially and this sample was incubated at 37 °C for 15 min. The pellet was washed twice with water. Finally, the pellet was resuspended in 2–4 ml of water and lyophilized, yielding the purified PGN-WTA complex. Depending on the specific research question, further chemical or enzymatic treatments were performed subsequently.

### Chemical and enzymatic treatments

*Hydrazine treatment of LTA:* Purified LTA was dissolved at a concentration of 5 µg µl^−1^ in anhydrous hydrazine (N_2_H_4_; ICN Biomedicals) before incubation for 1 h at 37 °C while being stirred. The reaction was quenched by adding the same volume of acetone and dried under a stream of nitrogen; the drying step was repeated twice. Subsequently, the crude de-*O*-acylated LTA was purified by gel permeation chromatography (GPC) on a Bio-Gel P-10 (45–90 µm, BioRad; column size: 1.5 × 120 cm; buffer: 150 mM ammonium acetate (pH 4.7)) column.

*Enzymatic digestion of the PGN-WTA complex:* To remove all amino acids from the PGN, the PGN-WTA complex was dissolved in 50 mM Tris-HCl (pH 7.0; 10 mg ml^−1^) and treated with the pneumococcal LytA amidase as described elsewhere^11^. Recombinant His-tagged LytA amidase (10 µg LytA per mg) was added in three aliquots after 0, 24, and 48 h for a total period of incubation of 72 h at 37 °C. Subsequently, the enzyme was inactivated by boiling for 5 min at 100 °C. After centrifugation (25,000×*g*, 15 min, 20 °C), the supernatant was collected and lyophilized. The crude LytA-treated PGN-WTA complex was further purified by GPC on a Bio-Gel P-30 (45–90 µm, BioRad; column size: 1.5 × 120 cm; buffer: 150 mM ammonium acetate (pH 4.7)) column. The high molecular weight material obtained (~12 mg) was further digested with lysozyme (200 µg; Sigma) and mutanolysin (200 µg; Sigma) in an 800 µl reaction mixture containing sodium phosphate (20 mM; pH 4.8) and sodium azide (0.02%) at 37 °C overnight. The enzymes were inactivated by heating at 100 °C for 5 min. The soluble material was recovered by centrifugation (18,000×*g*, 10 min, 20 °C) and lyophilized. Isolation of the pnWTA bound to small PGN fragments was achieved by a final GPC using the conditions mentioned above.

### NMR spectroscopy

NMR spectroscopic measurements were performed in D_2_O at 300 K on a Bruker Avance^III^ 700 MHz (equipped with an inverse 5 mm quadruple-resonance Z-grad cryoprobe). Deuterated solvents were purchased from Deutero GmbH (Kastellaun, Germany). Acetone was used as an external standard to calibrate ^1^H (δ_H_ 2.225) and ^13^C (δ_C_ 30.89) NMR spectra. ^31^P NMR spectra (δ_P_ 0.0) were calibrated with 85% phosphoric acid in D_2_O as an external standard. ^1^H NMR assignments were confirmed by two-dimensional ^1^H, ^1^H COSY and TOCSY experiments, and ^13^C NMR assignments were indicated by two-dimensional ^1^H, ^13^C HSQC, based on the ^1^H NMR assignments. Interresidual connectivity and further evidence for ^13^C assignment were obtained from two-dimensional ^1^H, ^13^C HMBC and ^1^H, ^13^C HSQC-TOCSY experiments. Phosphate group connectivity was assigned by two-dimensional ^1^H, ^31^P HMQC and ^1^H, ^31^P HMQC-TOCSY. All data were acquired and processed using Bruker TOPSIN V 3.0 or higher.

### Mass spectrometry

To analyze the pnWTA bound to small PGN fragments, electrospray ionization fourier-transform ion cyclotron resonance mass spectrometry (ESI-FT-ICR-MS) was performed on a 7 Tesla APEX Qe instrument (Bruker Daltonics, Bremen, Germany) using negative-ion mode and a water/propan-2-ol/7 M triethylamine/acetic acid mixture (50:50:0.06:0.02 v/v/v/v) as solvent, as described previously^6^. MS analysis of hydrazine-treated LTA was done on a Q Exactive Plus (Thermo Scientific, Bremen, Germany) in negative-ion mode using the same solvent. A Triversa Nanomate (Advion, Ithaca, USA) ion source was used with a spray voltage set to −1.1 kV. The mass scale was calibrated externally with glycolipids of known structure, and all spectra were charge deconvoluted. The given mass numbers refer to the monoisotopic mass of the neutral molecules.

### Electron microscopy

*Field emission scanning electron microscopy:* bacteria were fixed in the growth medium with 5% formaldehyde and 2.5% glutaraldehyde for 1 h on ice and washed with HEPES buffer (HEPES 0.1 M, 0.09 M sucrose, 10 mM CaCl_2_, 10 mM MgCl_2_, pH 6.9). An aliquot of 50 µl of the fixed bacterial solution was placed on poly-l-lysine coated coverslips and allowed to settle for 10 min. After fixation with 2% glutaraldehyde in PBS for 5 min at room temperature, the coverslips were washed with TE buffer (20 mM Tris-HCl, 1 mM EDTA, pH 6.9) before dehydrating in a graded series of acetone (10, 30, 50, 70, 90, and 100%) on ice for 10 min for each step. Samples in the 100% acetone step were allowed to reach room temperature before another change in 100% acetone before critical-point drying with liquid CO_2_ (CPD 30, Balzers, Liechtenstein). Dried samples were covered with a palladium–gold film by sputter coating (SCD 500, Bal-Tec, Liechtenstein) before examination in a field emission scanning electron microscope Zeiss Merlin (Oberkochen, Germany) using the HESE2 Everhart Thornley SE detector and the in-lens SE detector in a 25:75 ratio at an acceleration voltage of 5 kV.

*Transmission electron microscopy:* bacteria were fixed as above and further fixed with osmium tetroxide (1% in HEPES buffer) for 1 h at room temperature. After washing with HEPES buffer, samples were dehydrated with 10, 30, and 50% acetone on ice before incubation in 70% acetone with 2% uranyl acetate overnight at 7 °C. Samples were further dehydrated with 90 and 100% acetone on ice, allowed to reach room temperature and further dehydrated with 100% acetone, then changed into 100% ethanol. Subsequently, samples were infiltrated with the aromatic acrylic resin LRWhite. After polymerization for 2 days at 50 °C, ultrathin sections were cut with a diamond knife, collected onto butvar-coated 3000 mesh grids, and counterstained with 4% aqueous uranyl acetate for 3 min. Samples were imaged in a Zeiss TEM 910 at an acceleration voltage of 80 kV and at calibrated magnifications.

Contrast and brightness were adjusted with Adobe Photoshop CS3.

### Generation of antibodies

Polyclonal antibodies against analyzed CBPs were raised in mice using routine immunization protocols. Briefly, CD-1 mice were immunized intraperitoneally with 20 µg recombinant protein and Freund's incomplete adjuvant (Sigma-Aldrich, Darmstadt, Germany) (50:50 v/v). At day 14 and 28, mice were boosted with 20 µg protein and Freund's incomplete adjuvant (50:50 v/v). Mice were bled at day 42 and polyclonal IgG were purified from serum using protein A-Sepharose (Sigma-Aldrich, Darmstadt, Germany). Antibodies are listed in Supplementary Table 2.

### Flow cytometry

*S. pneumoniae* D39 wild type, its isogenic *tacL* mutant, and the complemented mutant were cultivated in THY medium to mid-log phase, harvested at 3,275×*g* for 6 min, and washed with PBS (pH 7.4). For quantification of the capsule content, a 100 µl suspension containing 4 × 10^8^ bacteria was incubated with anti-capsular polysaccharide antisera (SSI Type serum 2, Statens Serum Institute) (1:500 in PBS) for 30–45 min at 37 °C and 5% CO_2_ in 96-well plates (U-bottom, Greiner Bio-One). After washing, samples were incubated with a secondary goat anti-rabbit IgG-coupled Alexa_488_-labeled antibody (Invitrogen) (1:500 in PBS, 30–45 min, 37 °C). After washing with PBS, bacteria were fixed with 1% formaldehyde overnight at 4 °C.

The abundance of CBPs as well as the quantity of teichoic acids in nonencapsulated D39 wild-type, mutant and complemented strains were measured by flow cytometry after fixation with 1% formaldehyde for 1 h at 4 °C. Two hundred µl suspensions containing 8 × 10^8^ bacteria were incubated with specific polyclonal antibodies against different CBPs (1:500 in PBS), or for the quantification of TAs with antibodies against *P*-Cho (TEPC-15) or Forssman antigen (15 min at 37 °C and 5% CO_2_) in 96-well plates (U-bottom, Greiner Bio-One). After washing, samples were incubated with a secondary goat anti-IgG-coupled Alexa_488_-labeled antibody (Invitrogen) (15 min, 37 °C). Fluorescence was determined using a FACS Calibur™ (BD Biosciences).

### Immunoblot analysis

*S. pneumoniae* D39, its isogenic *tacL* mutant, and the complemented mutant were grown in THY medium until an *A*_600_ of 0.35–0.45 was reached, harvested by centrifugation at 3270×*g* at 4 °C for 6 min, and resuspended in 1 ml PBS buffer at pH 7.4. A total of 2 × 10^8^ cells per well were loaded and run on a 12% SDS-PAGE before transferring to a nitrocellulose membrane by semidry blotting. The membranes were blocked for 2 h at room temperature using 5% skimmed milk (Roth) and Tris-buffered saline (TBS; pH 7.4) and incubated overnight at 4 °C with mouse polyclonal antibodies against different CBPs (1:500 in 5% skimmed milk + TBS 0.01% Tween (T-TBS)). A rabbit polyclonal antibody against enolase (1:25,000 in 5% skimmed milk + T-TBS) was used as a loading control. The membranes were washed with T-TBS, CBPs were detected with the secondary fluorescence-labeled IRDye^®^ 800CW. Goat α-mouse IgG and enolase were detected with the fluorescence-labeled IRDye^®^ 680RD. Goat α-rabbit IgG antibody was detected by incubation with the appropriate antibody (1:15,000 in 5% skimmed milk in T-TBS) for 45 min in the dark at room temperature; the membranes were washed with T-TBS and finally once with TBS. The scanning of the membranes was performed using an Odyssey^®^ CLx (LI-COR) scanner.

### Triton X-100-induced autolysis assay

*S. pneumoniae* D39 wild-type, mutant, and complemented strain were cultivated in THY medium to mid-log phase, harvested at 3275×*g* for 6 min, and washed with PBS (pH 7.4). A 1 ml suspension containing 1 × 10^9^ bacteria was incubated with a final concentration of 0.01% Triton X-100 (Sigma-Aldrich, Darmstadt, Germany) and incubated at 37 °C. Bacterial cell lysis was monitored by measuring absorbance at 600 nm at predefined time points.

### Epithelial adherence assay

Pneumococcal adherence to epithelial cells was analyzed with human A549 cells (ATCC^®^ CCl-185^TM^) as described^48^. Briefly, confluent epithelial cells, grown on glass coverslips (Hartenstein; in 24-well plates, ~1 × 10^5^ cells per well) were inoculated with 5 × 10^6^ mid-exponentially grown pneumococci and incubated in infection medium (DMEM (HyClone™) + 1% heat-inactivated fetal bovine serum (FBS)) at 37 °C and 5% CO_2_. Subsequently, cells were washed three times with phosphate-buffered saline containing 1% FBS (Gibco) to remove unbound bacteria. Afterwards, bacteria were fixed with PBS containing 1% para-formaldehyde (PFA, Roth).

### Immunofluorescence microscopy

Fixed pneumococci, bound to A549 cells were washed three times with PBS and blocked for 1 h at room temperature using PBS + 10% FBS. After washing, samples were incubated for 1 h at room temperature with a polyclonal antibody (1:500, Davids Biotechnologie GmbH) against pneumococci (generated in rabbit against heat-inactivated *S. pneumoniae* TIGR4 and D39). As a secondary antibody, fluorescence-labeled Alexa-Fluor_488_ goat anti-rabbit antibody (Abcam) was used (1:500, 1 h, room temperature). Bacterial adherence was monitored for at least 20 cells per class coverslip using fluorescence microscopy. Each experiment was repeated three times in duplicate. All data are reported as means ± s.d. Statistical analysis was performed using the unpaired two-tailed Student’s *t* test. In all analysis, a *p* value of <0.05 was considered statistically significant.

### Phagocytosis experiments

*Antibiotic protection assay*: A confluent layer of monocytic THP-1 cells (ATCC^®^ TIB-202^TM^) grown in 24-well plates (3 × 10^5^ cells per well in RPMI-1640 (HyClone™) supplemented with 10% heat-inactivated fetal bovine serum (FBS, Gibco)) was differentiated to phagocytes by the addition of 200 nmol ml^−1^ phorbol 12-myristate 13-acetate (PMA, Sigma-Aldrich) and incubated for 48 h at 37 °C and 5% CO_2_. Afterwards, THP-1 cells were washed with RPMI-1640 supplemented with 10% heat-inactivated FBS and incubated for another 24 h at 37 °C and 5% CO_2_. Prior infection pneumococci were cultured in THY to mid-log phase (*A*_600_ = 0.35–0.45), centrifuged, and washed with infection medium (RPMI-1640 supplemented with 1% heat-inactivated FBS). THP-1 cells were washed and infected with pneumococci in 500 µl infection medium. Infection was synchronized by centrifugation (2 min, 300×*g*) to initiate a simultaneous contact between bacteria and phagocytes. Afterwards cells were incubated at 37 °C and 5% CO_2_ for different time periods. After infection, cells were washed with infection medium and incubated with Penicillin G (100 unit ml^−1^, Sigma-Aldrich) and Gentamicin (0.1 mg ml^−1^, Sigma-Aldrich) for 1 h at 37 °C and 5% CO_2_ to kill extracellular bacteria. Then, phagocytes were washed and lysed with 1% saponin (Sigma-Aldrich) to release intracellular pneumococci. Colony-forming units (cfu) of intracellular bacteria were determined by plating bacteria in appropriate dilutions on blood agar plates (Oxoid)^49^. Time-dependent killing of intracellular pneumococci was evaluated by killing extracellular pneumococci using antibiotics as described above. Afterwards, phagocytes were further incubated in infection medium for different time periods (0–3 h). Intracellular bacterial cfu were monitored as described above. All experiments were repeated four times as duplicates. Data were normalized in the antibiotic protection assays to the multiplicity of infection (MOI) or in the time-dependent killing to recovered bacteria at time point 0 h. All assays were analyzed using one-way ANOVA with Bonferroni correction.

### Double immunofluorescence staining and microscopy

PMA-differentiated THP-1 cells (3 × 10^5^ cells per well) were grown on sterile glass coverslips (12 mm, Hartenstein) and infected with pneumococci as described above. After infection, THP-1 cells were washed with infection medium and fixed with 4% para-formaldehyde (Roth) over night at 4 °C. Glass coverslips were washed with PBS and blocked with PBS + 10% heat-inactivated FBS for 1 h. After washing, extracellular bacteria were stained using a polyclonal α-pneumococci IgG (1:500) and an Alexa-Fluor_488_-labeled secondary goat α-rabbit IgG (1:500, Abcam) for 30 min at room temperature. Glass coverslips were washed thrice with PBS following permeabilization of THP-1 cells with 0.1% Triton X-100 in PBS (10 min, room temperature). After washing, intracellular pneumococci were stained using a polyclonal α-pneumococci IgG (1:500) and a secondary Alexa-Fluor_568_-labeled goat α-rabbit IgG (1:500, Abcam) for 30 min at room temperature. Experiments were repeated three times as duplicates. For statistical analysis, 50 cells per glass coverslip were analyzed for the number of intracellular bacteria. Data were normalized to the MOI. All assays were analyzed using one-way ANOVA with Bonferroni correction. In all analysis, a *p* value of <0.05 was considered statistically significant.

### Cell lines

All cell lines used in this study were purchased from ATCC (A549: ATCC CCL-185; THP-1: ATCC TIB-202) and have been tested to be mycoplasma-negative by PCR and scanning electron microscopy.

### Acute mouse pneumonia and systemic infection model

Eight- to 10-week-old female CD-1 mice (outbred, Charles River, Sulzfeld, Germany) were intranasally infected with bioluminescent pneumococci as described recently^50^. Briefly, pneumococci were cultured to mid-exponential phase (*A*_600_ = 0.35) in THY medium containing 10% heat-inactivated fetal bovine serum. After centrifugation, the infection dose (20 µl) was adjusted to ~2.5 × 10^7^ cfu in PBS (pH 7.4). For nasal infection, mice were anesthetized by intraperitoneal injection of ketamine/xylazine (Ketanest S, Pfizer Pharma, Karlsruhe, Germany; Rompun, Provet AG, Lyssach, Germany), and bacteria were administered dropwise into the nostrils. The cfu of the infection dose was confirmed by plating of serial dilutions of the inoculum on blood agar plates. Mice were monitored for survival and imaged for bioluminescence at pre-chosen intervals using the IVIS^®^ Spectrum Imaging System (Caliper Life Sciences, Hopkinton, USA). For the systemic infection model, an infection dose of 3 × 10^3^ cfu was administered intraperitoneally in a volume of 200 µl PBS (pH 7.4). All animal experiments were conducted according to the German regulations of the Society for Laboratory Animal Science (GV-SOLAS) and the European Health Law of the Federation of Laboratory Animal Science Associations (FELASA). All experiments were approved by the Landesamt für Landwirtschaft, Lebensmittelsicherheit und Fischerei Mecklenburg – Vorpommern (LALLFV M-V, Rostock, Germany, permit no. 7221.3-1-056/16-1).

### Data availability

Raw FASTQ files containing *S. pneumoniae* genomic sequence data were submitted to the EMBL-EBI European Nucleotide Archive (ENA) and stored in the Short Read Archive (SRA) under the study accession number RJEB18558. All other relevant data supporting the findings of the study are available in this article and its Supplementary Information files, or from the corresponding authors on request.

## Electronic supplementary material

Supplementary Information
