# Supplementary material for: Lipoteichoic acid deficiency permits normal growth but impairs virulence of Streptococcus pneumoniae
Source: Nat Commun. 2017 Dec 12;8:2093. doi: 10.1038/s41467-017-01720-z (PMC5727136; doi:10.1038/s41467-017-01720-z)
Supplement: Supplementary file 1 — Supplementary Information [file 41467_2017_1720_MOESM1_ESM.pdf]

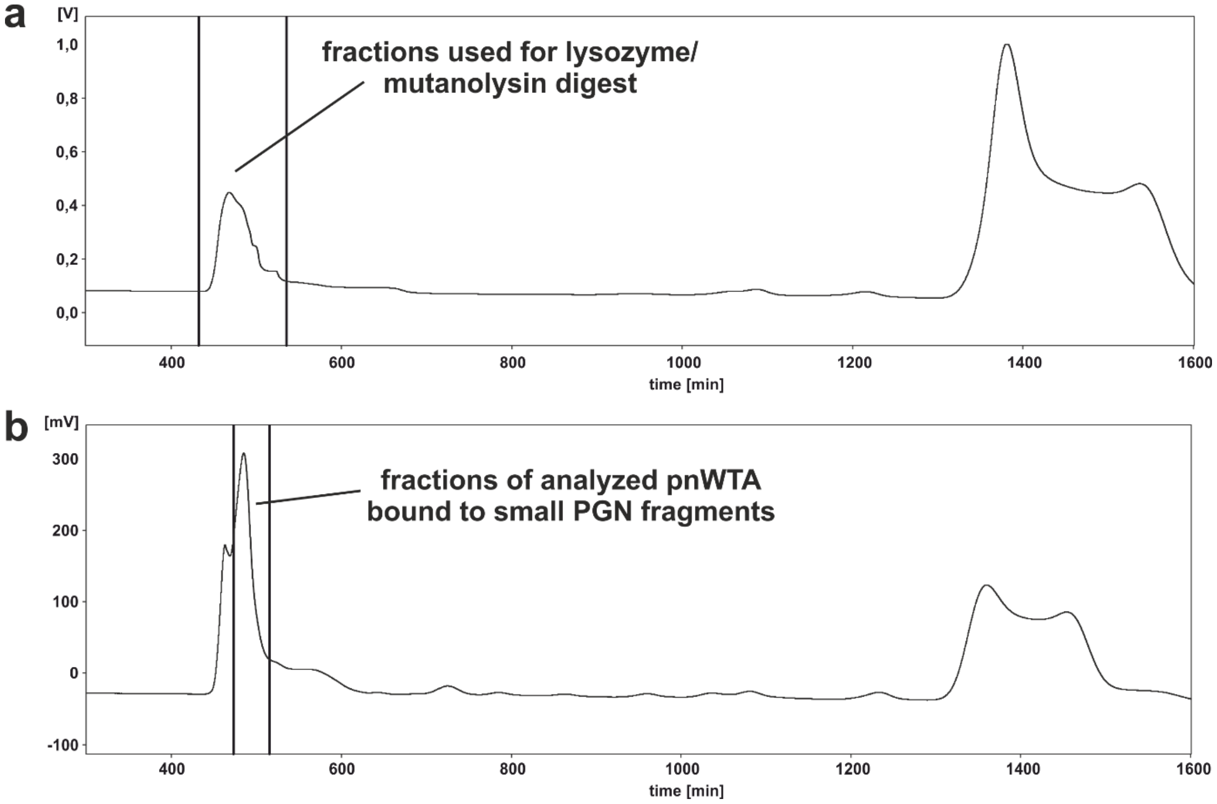

Supplementary Figure 1. Gel permeation chromatography for the isolation of pnWTA bound to small PGN derived saccharides from *S. pneumoniae* D39Δ*cps*Δ*lgt*. Chromatogram of the GPC (Bio-Gel P-10 column, 150 mM ammonium acetate (pH 4.7)) of (**a**) the pnWTA-PGN complex after LytA-treatment. The indicated fractions were further used for lysozyme/mutanolysin digestion, which yield after a second GPC (**b**; same conditions as in (**a**)) a portion of pnWTA bound to small PGN derived saccharides, whose analysis is depicted in Fig. 1a-c and Supplementary Fig. 2.

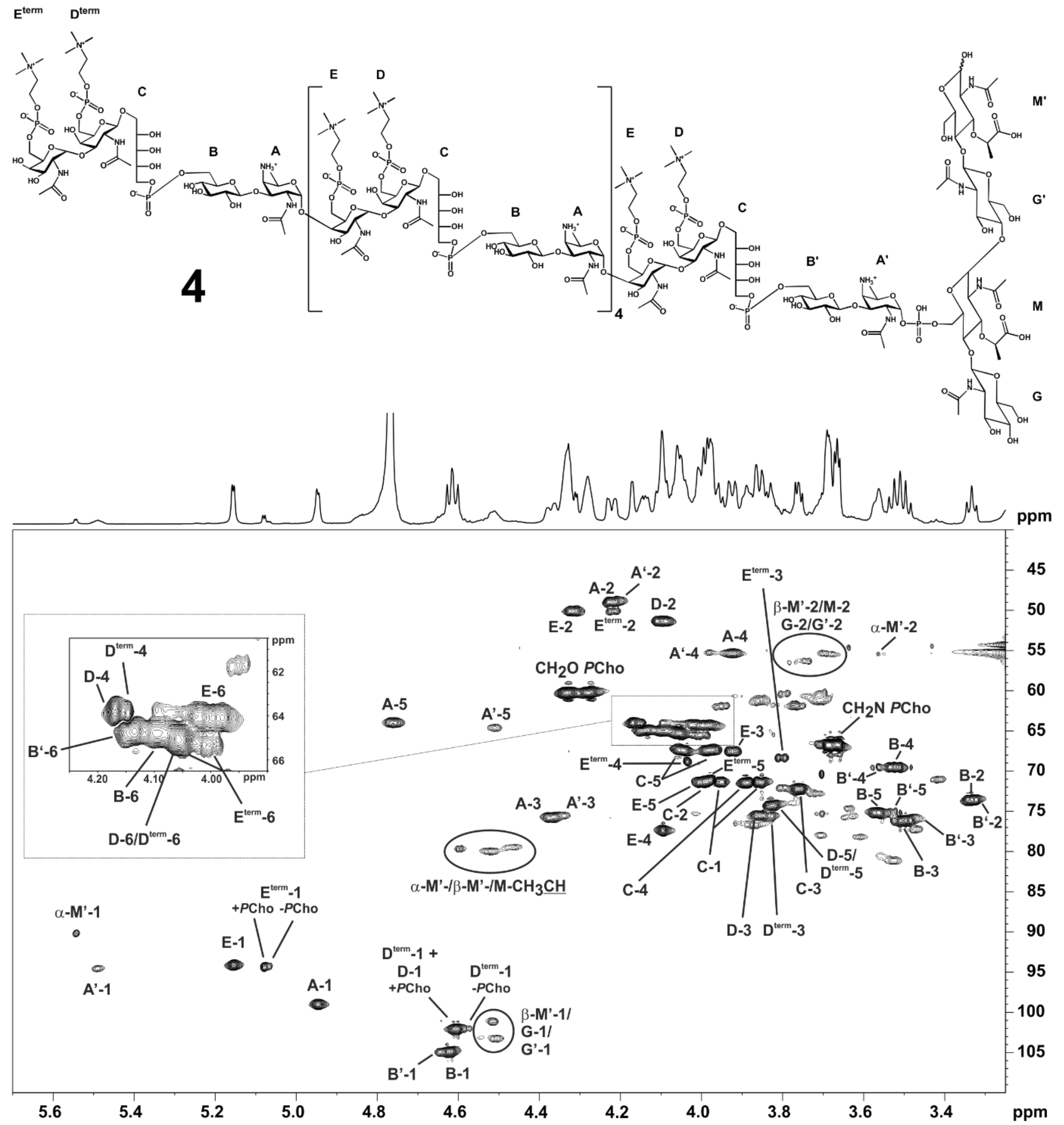

Supplementary Figure 2. Section (δ_H 5.70-3.25; δ_C 110-40) of the ^1H, ^{13}C-HSQC NMR spectrum (700 MHz) obtained from pnWTA bound to small PGN saccharides isolated from *S. pneumoniae* D39Δ*cps*Δ*lgt*. Mass spectrometric and further NMR analysis of the used material is depicted in Figs 1a-c. Detailed NMR chemical shift data are listed in Supplementary Table 1. Assignment of signals is exemplified on the structure of **4**, which was the major component in this preparation as identified by MS (Fig. 1a). Only a few PGN-derived signals (G, G', M, M') could specifically be assigned due to microheterogeneity of PGN part structures and their low intensity compared to pnWTA signals.

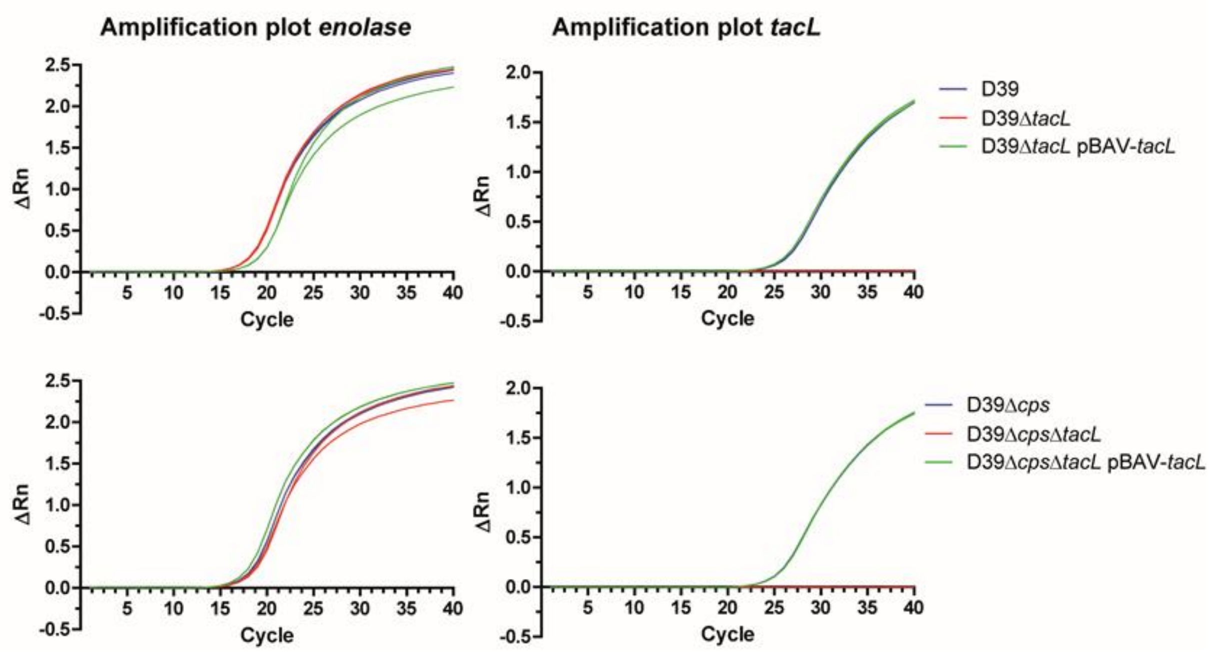

Supplementary Figure 3: Real-time quantitative PCR (qRT-PCR). To verify the *tacL* deletion and complementation**,** encapsulated and nonencapsulated *S. pneumoniae* D39, D39Δ*tacL* and the complemented D39Δ*tacL* mutant were grown in THY until mid-log phase. After RNA-isolation and cDNA synthesis using random DNA hexamer primers, *tacL* was amplified by PCR using primer qP_tacL_F and qP_tacL_R (see Supplementary Table 2). Each strain was analyzed in duplicate (2 lines per strain in the diagram). The *enolase* gene was used as control.

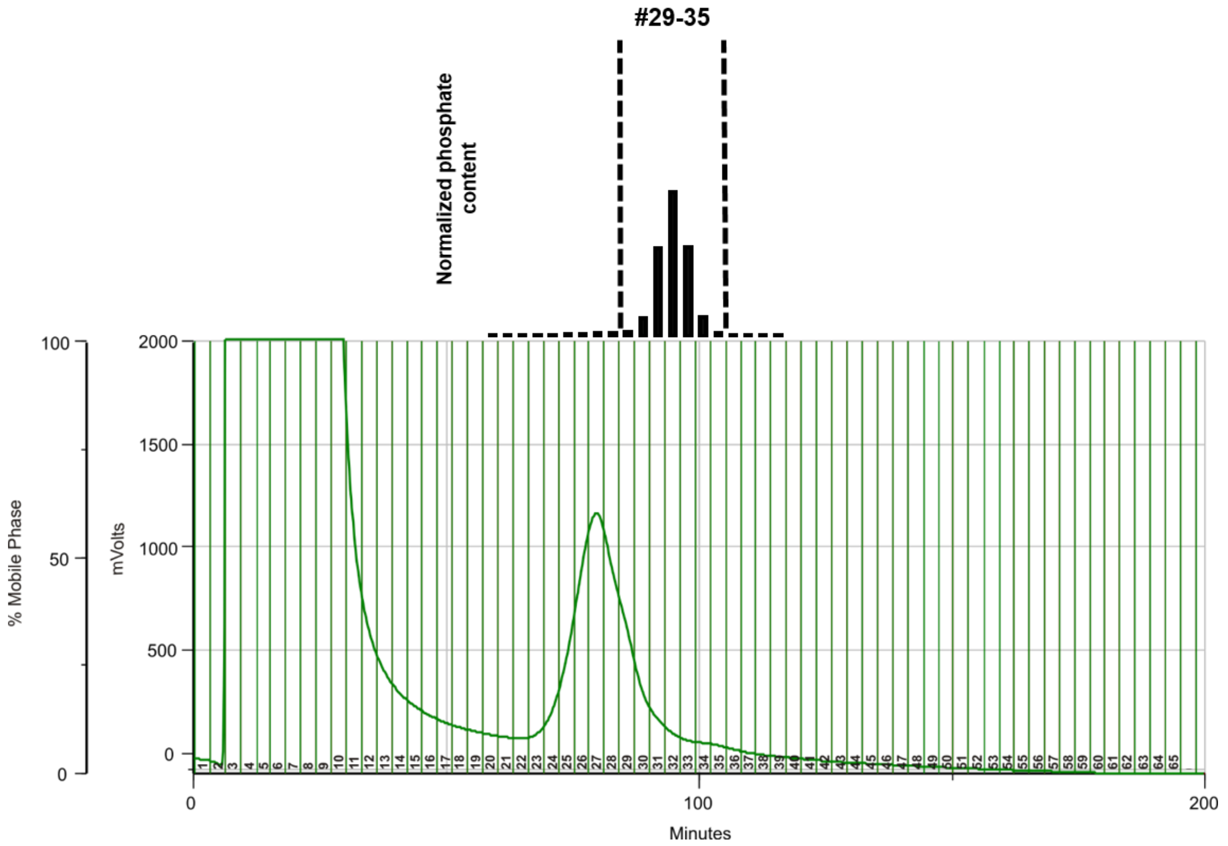

Supplementary Figure 4. Representative chromatogram of the hydrophobic interaction chromatography (HIC) of D39Δ*cps* LTA including normalized visualization of the phosphate content of the selected fractions. The fractions #29-35 were combined on the basis of the phosphate content and contain the LTA. The main UV active signals in fractions #23-40 are caused by non-LTA species such as lipoproteins. For *tacL* mutants the phosphate content of these fractions was zero. However, for ^{31}P NMR measurements shown in Figs 2b,e the usually LTA containing fractions were pooled and used.

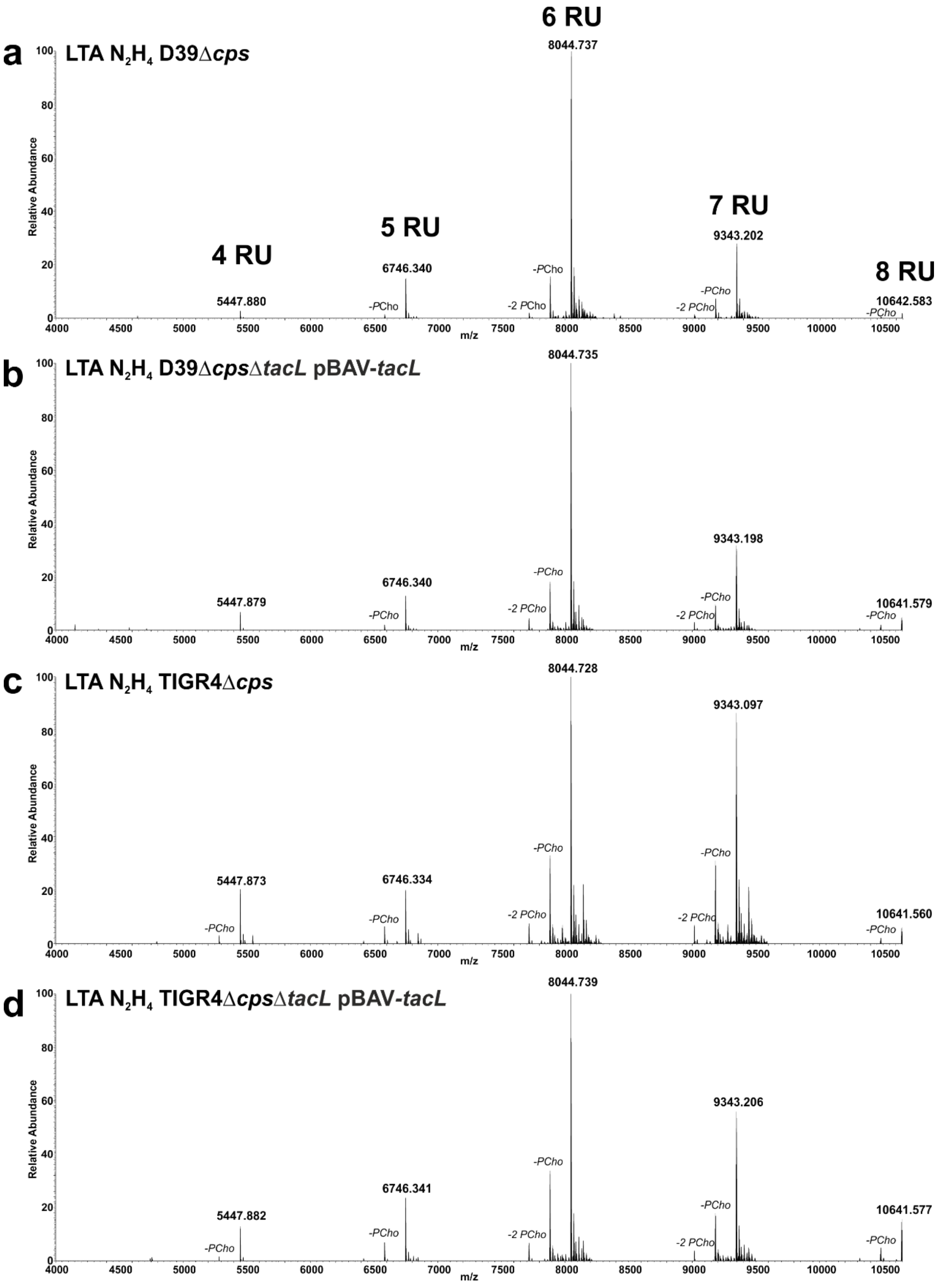

Supplementary Figure 5. Section of charge deconvoluted mass spectra (acquired in negative ion mode) of hydrazine-treated LTA of *S. pneumoniae* LTA. Shown are the mass spectra for pnLTA after hydrazine treatment isolated from strain (**a**) D39Δ*cps*, (**b**) D39Δ*cps*Δ*tacL* pBAV-*tacL*, (**c**) TIGR4Δ*cps*, and (**d**) TIGR4Δ*cps*Δ*tacL* pBAV-*tacL*. The monoisotopic mass of 5447.889 Da (calculated) corresponds to de-*O*-acylated pnLTA with n = 2 (structure shown in Fig. 1d), the assigned higher masses to respective molecules with longer chains (6746.336 Da (n = 3); 8044.783 Da (n = 4); 9343.231 Da (n = 5); 10641.678 Da (n = 6)).

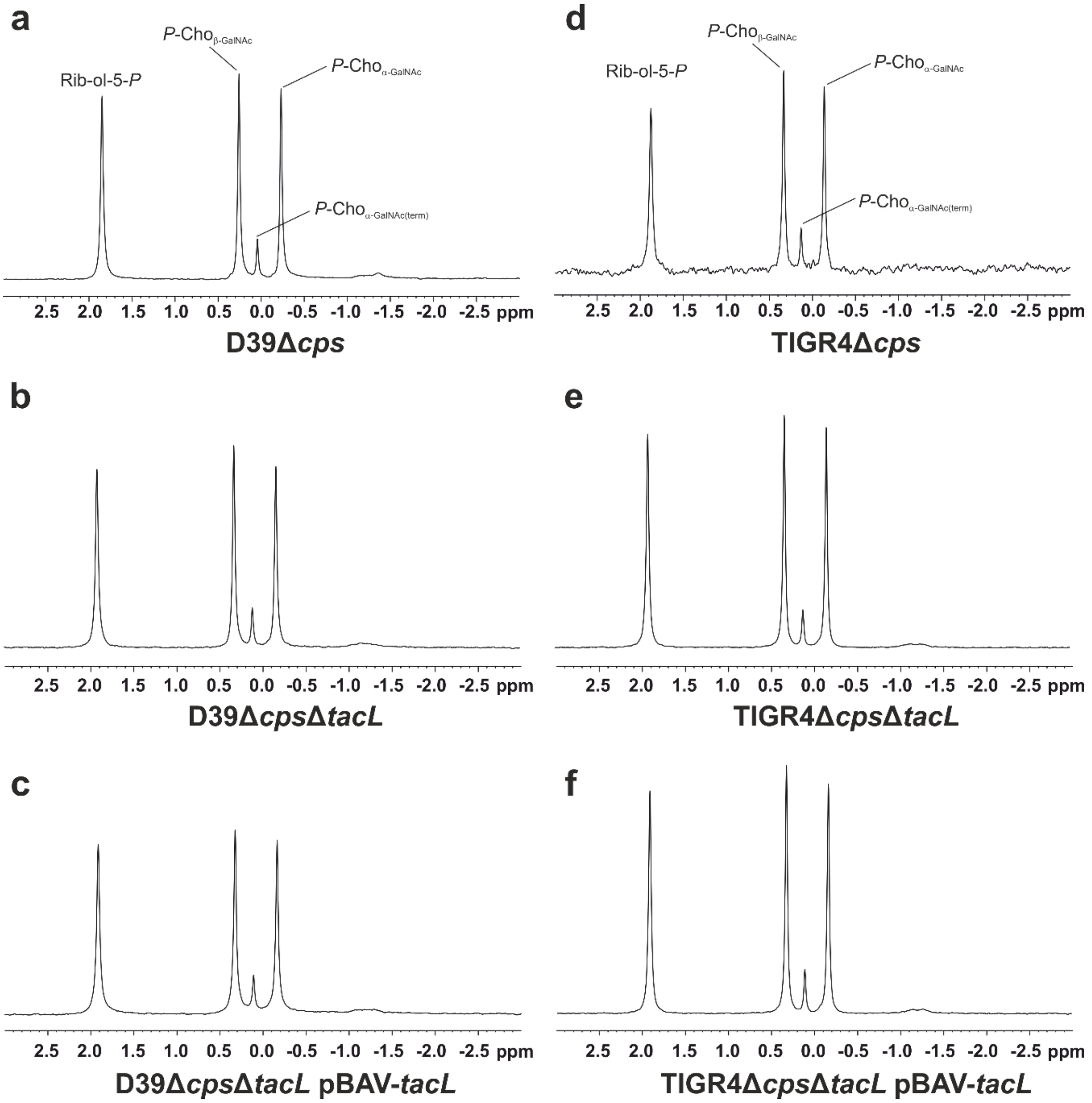

Supplementary Figure 6. ^{31}P NMR spectra of LytA-treated PGN-pnWTA complex. Sections of ^{31}P NMR (δ_P (3-(-3)) of the PGN-pnWTA complex after LytA-treatment and GPC purification isolated from D39 strains (**a**) D39Δ*cps*, (**b**) D39Δ*cps*Δ*tacL*, (**c**) D39Δ*cps*Δ*tacL* pBAV-*tacL* and TIGR4 strains (**d**) TIGR4Δ*cps*, (**e**) TIGR4Δ*cps*Δ*tacL* and (**f**) TIGR4Δ*cps*Δ*tacL* pBAV-*tacL*.

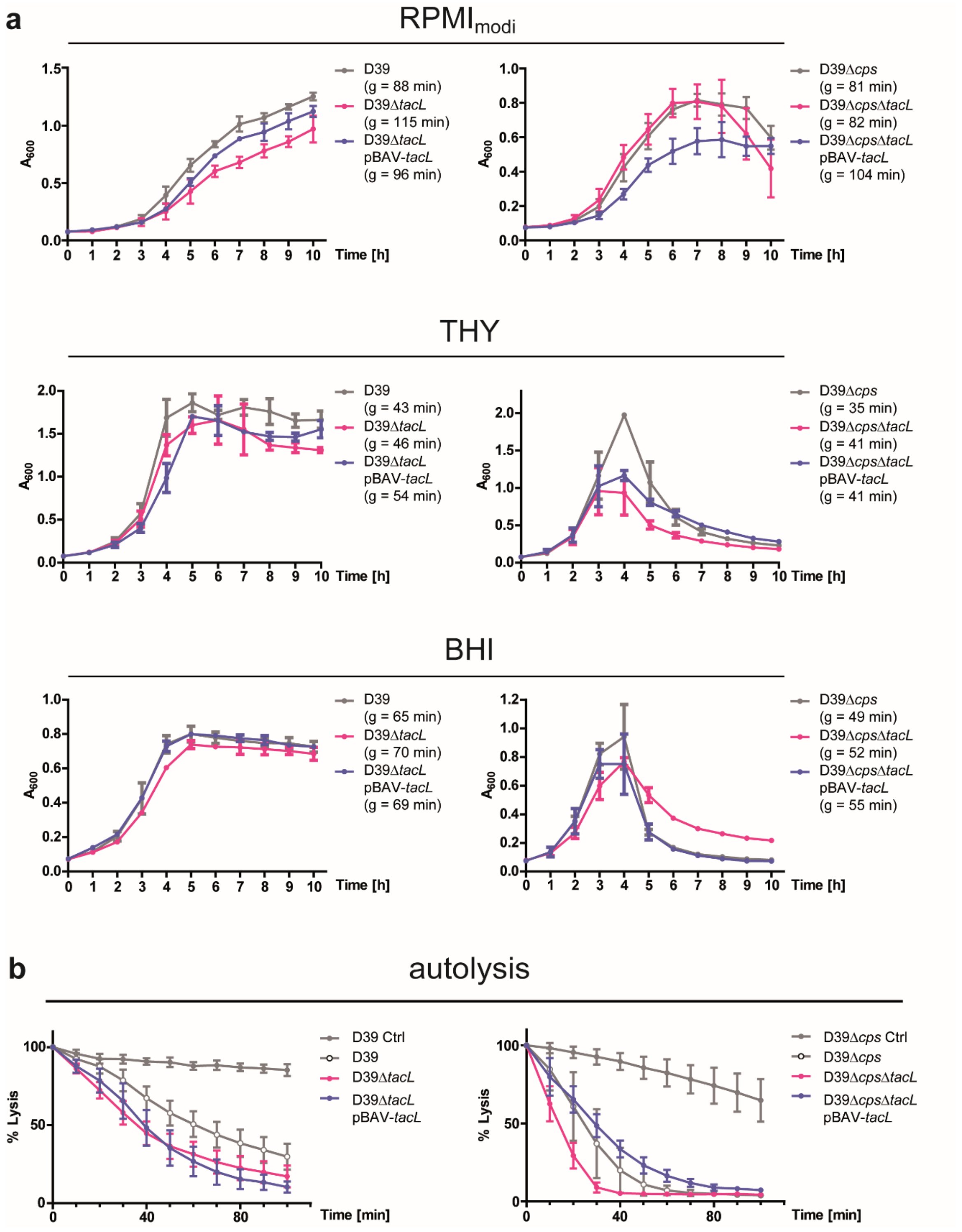

Supplementary Figure 7: Pneumococcal growth curves and autolysis. (**a**) Growth curves of nonencapsulated (Δ*cps*) *S. pneumoniae* D39 and encapsulated D39 wild-type, isogenic Δ*tacL* mutant as well as complemented mutant (Δ*tacL* pBAV-*tacL*) strains in chemically-defined medium ($RPMI_{modi}$) and complex media (Todd-Hewitt broth + 0.5% yeast (THY) or brain heart infusion broth (BHI)). Absorbance at 600 nm was measured at different time points. g = generation time. (**b**) For the Triton X-100 induced autolysis assay, pneumococci were grown to mid-log phase, harvested and resuspended in PBS containing 0.01% Triton X-100 (A_{600} = 1). Bacterial lysis was monitored by measuring the absorbance at 600 nm. Error bars represent means ± s.d. of at least three independent experiments.

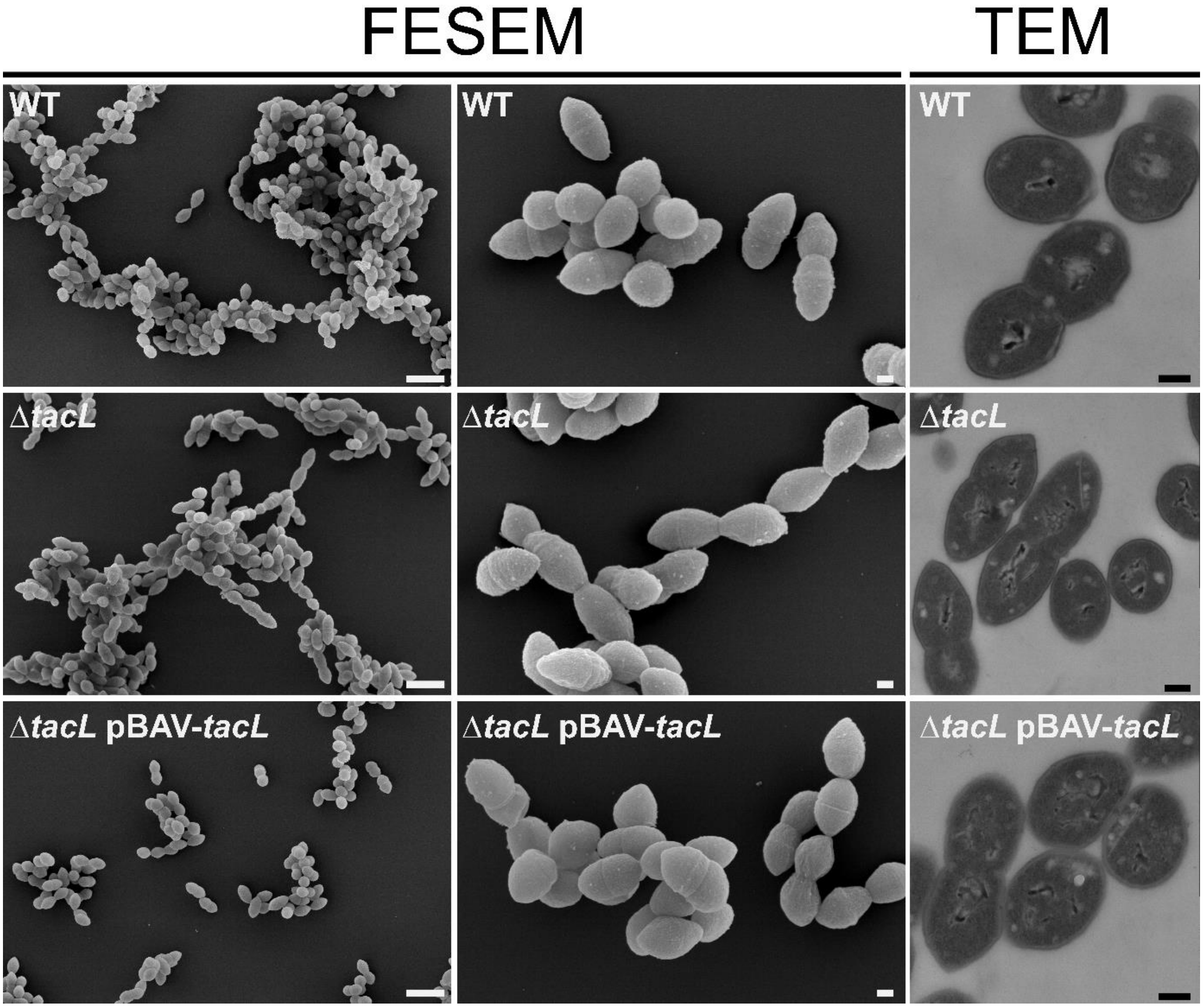

Supplementary Figure 8: Analysis of pneumococcal cell morphology and cell division by electron microscopy. FESEM and TEM ultrathin sections revealed no visible differences in the cell morphology and cell division septa morphology between nonencapsulated D39 wild-type (WT), *tacL* mutant (Δ*tacL*) or complemented mutant (Δ*tacL* pBAV-*tacL*) grown in chemically-defined medium ($RPMI_{modi}$). Scale bars = 2 µm (FESEM, left panel), 0.2 µm (FESEM, right panel), 0.2 µm (TEM).

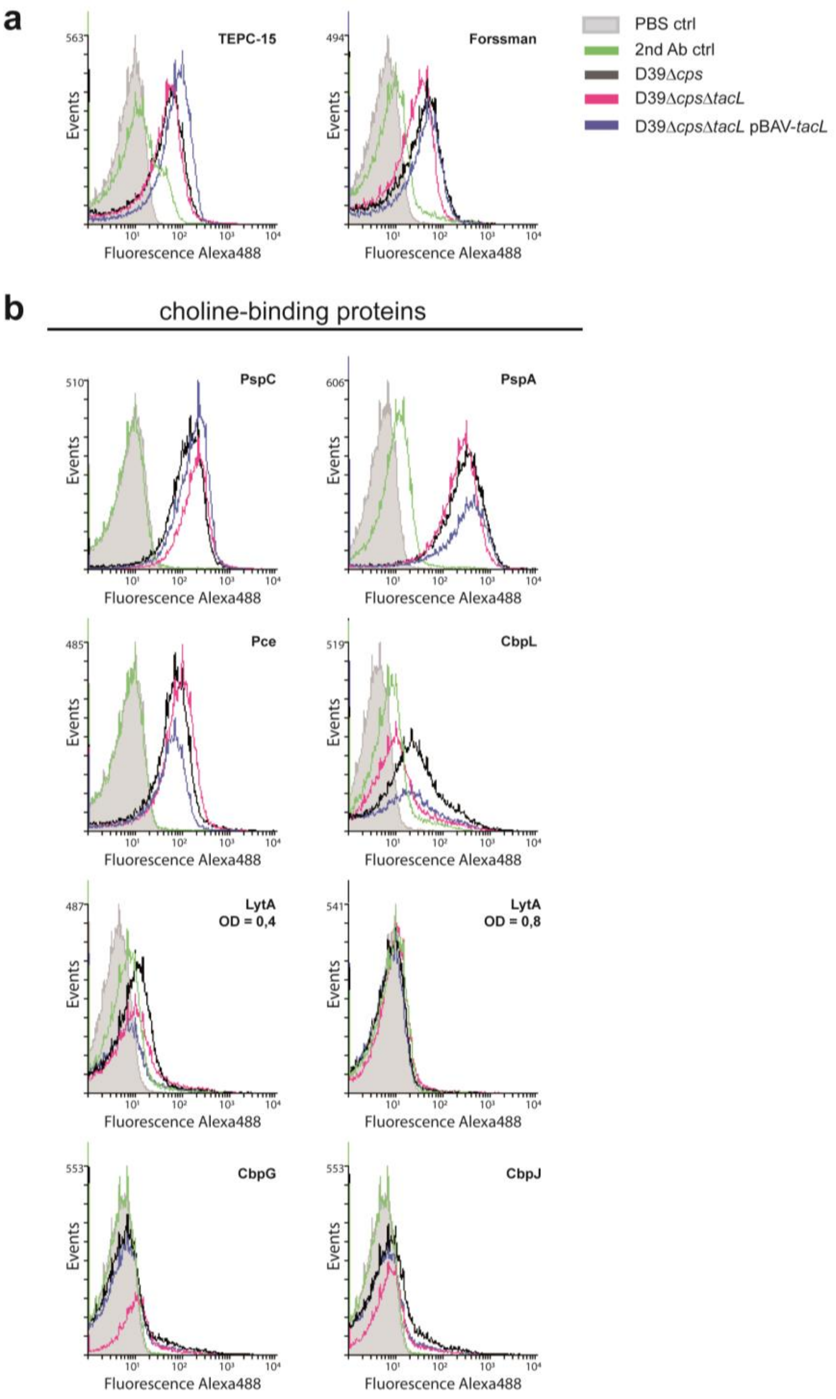

Supplementary Figure 9: Representative histograms of the quantitative analysis of teichoic acids and choline-binding proteins. Bacteria were grown in THY-medium to A_{600} = 0.35-0.45 (for LytA 0.4 and 0.8), washed and incubated with antibodies. (**a**) The amount of *P*-Cho and Forssman antigen of teichoic acids were determined using specific primary antibodies (TEPC-15, anti-Forssman) and secondary $Alexa_{488}$-labeled antibody in a flow cytometry based assay. (**b**) Surface associated CBPs were analyzed using specific polyclonal mice IgG against the individual CBPs and secondary $Alexa_{488}$-labeled antibodies in a flow cytometry based assay.

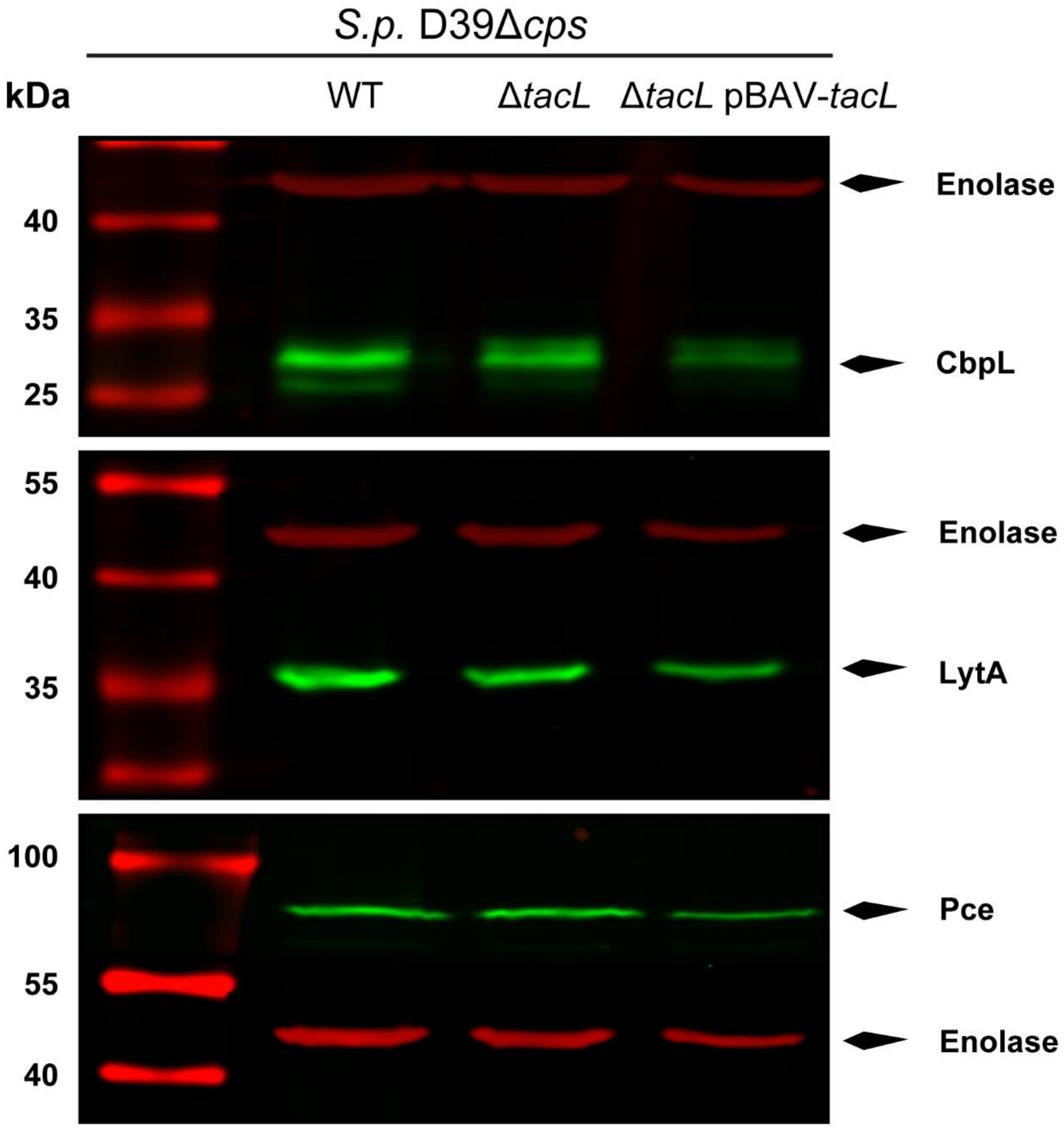

Supplementary Figure 10: Expression analysis of choline-binding proteins. Bacteria were grown in THY to A_{600} = 0.35-0.45, harvested by centrifugation and lysates were resuspended in PBS. 2 × 10^8 cells/well were loaded for each strain for SDS-PAGE and surface associated CBPs were analyzed by immunoblot using specific polyclonal mice IgG against single proteins and secondary fluorescence labeled IRDye® 800CW (LI-COR) Goat α-mouse IgG antibody. Specific polyclonal rabbit IgG against Enolase and secondary fluorescence labeled IRDye® 680RD (LI-COR) Goat α-rabbit IgG antibody were used as loading control.

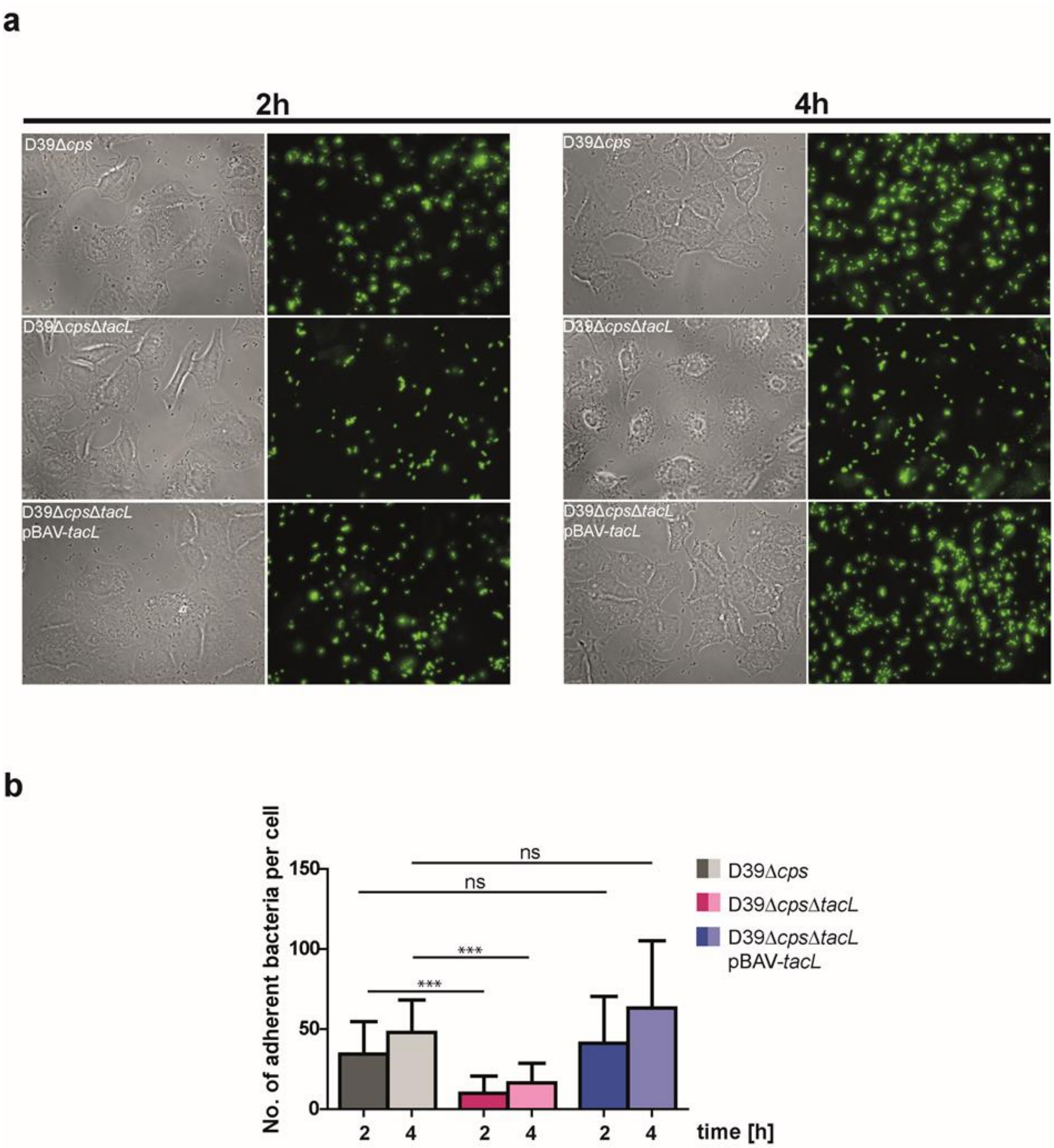

Supplementary Figure 11: Pneumococcal adherence to epithelial cells. (a) Fluorescence microscopy of adhered *S. pneumoniae* D39Δ*cps*, D39Δ*cps*Δ*tacL* or D39Δ*cps*Δ*tacL* pBAV-*tacL* to A549 human lung epithelial cells. Approximately 1×10^5 cells were infected with 5×10^6 pneumococci and incubated for 2 h or 4 h. Bacteria were stained using a polyclonal antibody (1:500) against pneumococci and a secondary antibody (fluorescence-labeled Alexa Fluor$_{488}$, 1:500). Magnification 630x. **(b)** Adhered bacteria were counted by immunofluorescence microscopy (20 cells/ glass coverslip). Values represent means ± s.d. of at least three independent experiments. ns = not significant, ***p < 0.001 (Unpaired two-tailed Student´s t test).

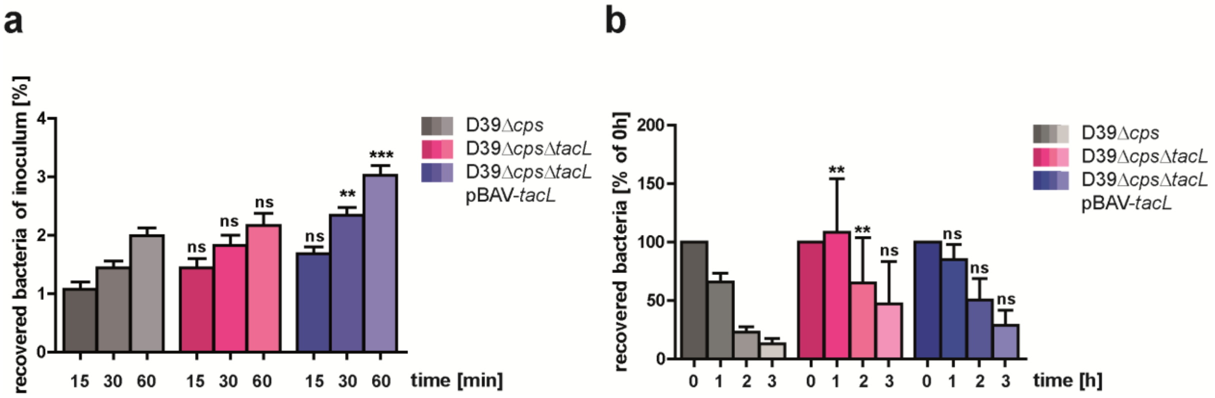

Supplementary Figure 12: Pneumococcal infection of human THP-1 cells. (a) Bacterial uptake by phagocytes. PMA-differentiated THP-1 cells were infected with nonencapsulated *S. pneumoniae* D39Δ*cps*, D39Δ*cps*Δ*tacL* or D39Δ*cps*Δ*tacL* pBAV-*tacL*. Post infection, extracellular pneumococci were killed at pre-chosen time points by the addition of Gentamicin and Penicillin and living intracellular bacteria were recovered and enumerated by plating of cell lysates on blood agar plates. **(b) Time-dependent killing of intracellular pneumococci.** To visualize the killing of intracellular pneumococci, THP-1 cells were infected for 60 minutes. Extracellular pneumococci were killed by antibiotics and the incubation was continued in infection medium without antibiotics. At different time-points post infection, the number of intracellular cfu was determined by lysis of the THP-1 cells and plating of cell lysates on blood agar plates. Values represent means ± s.d. of at least three independent experiments. ns = not significant, **$p < 0.01$; ***$p < 0.001$ (One-way Anova with Bonferroni correction).

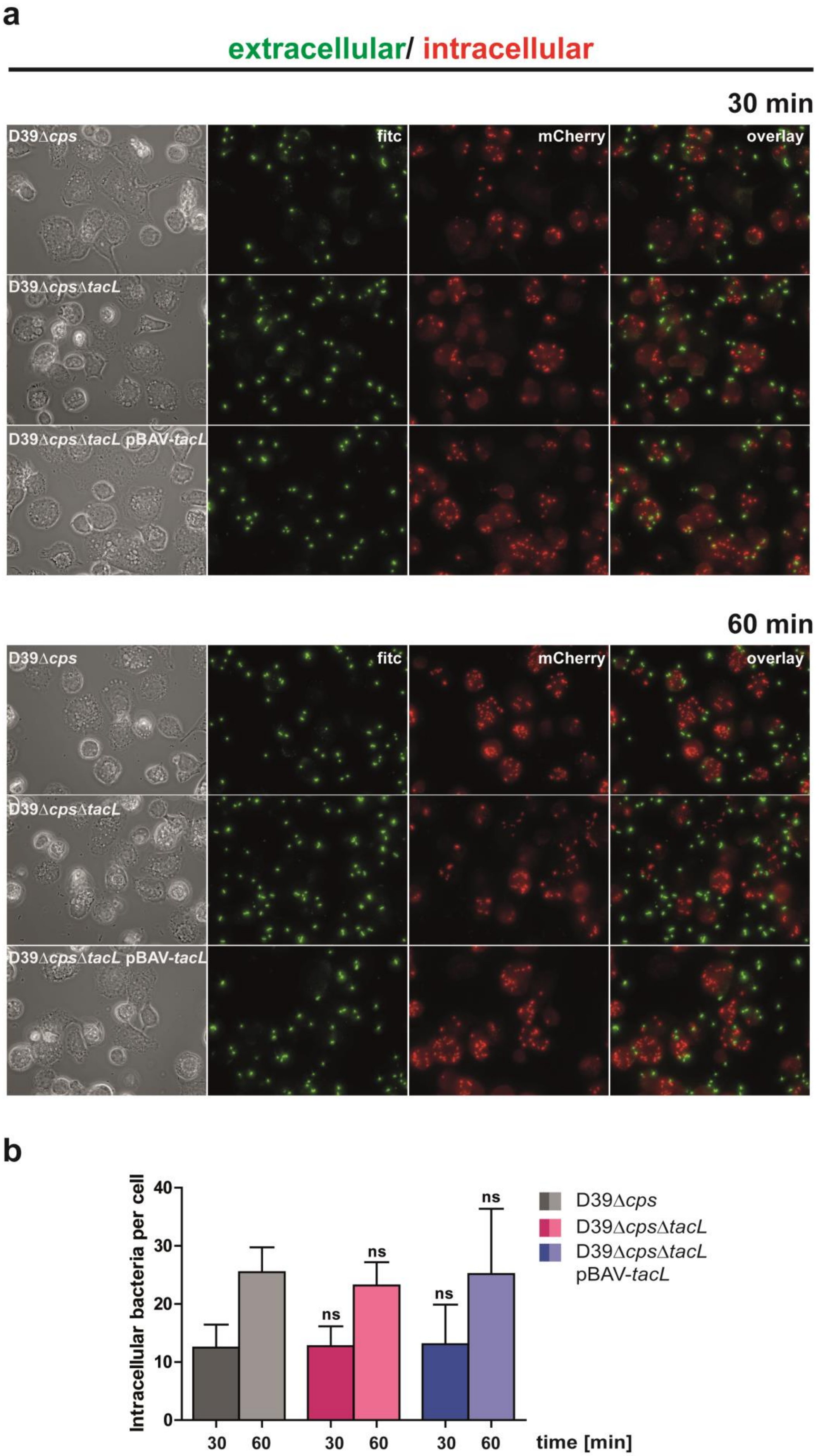
a
extracellular/ intracellular
30 min
D39Δcps
fitc
mCherry
overlay
D39ΔcpsΔtacL
D39ΔcpsΔtacL pBAV-tacL
60 min
D39Δcps
fitc
mCherry
overlay
D39ΔcpsΔtacL
D39ΔcpsΔtacL pBAV-tacL
b
Intracellular bacteria per cell
40
30
20
10
0
ns
ns
ns
ns
30
60
30
60
30
60
time [min]
D39Δcps
D39ΔcpsΔtacL
D39ΔcpsΔtacL pBAV-tacL

Supplementary Figure 13: Bacterial uptake by phagocytes and double immunofluorescence staining. (a) Time-dependent uptake of nonencapsulated *S. pneumoniae* D39Δ*cps*, D39Δ*cps*Δ*tacL* or D39Δ*cps*Δ*tacL* pBAV-*tacL* by PMA-differentiated THP-1 cells was monitored at different time points using double immunofluorescence staining. Extracellular pneumococci were stained using a polyclonal anti-pneumococcal antibody and Alexa $Fluor_{488}$ labeled secondary antibody (1:500, green, measured using the fitc channel). After permeabilization of THP-1 cells using 0.1% Triton-X 100, intracellular bacteria (red) were labeled using polyclonal anti-pneumococcal antibody and Alexa $Fluor_{568}$ labeled secondary antibody (1:500, measured using the mCherry channel). Magnification 630x. **(b)** Intracellular bacteria were counted by immunofluorescence microscopy (50 cells/ glass coverslip). Values represent means ± s.d. of at least three independent experiments. ns = not significant, *$p < 0.05$ (One-way Anova with Bonferroni correction).

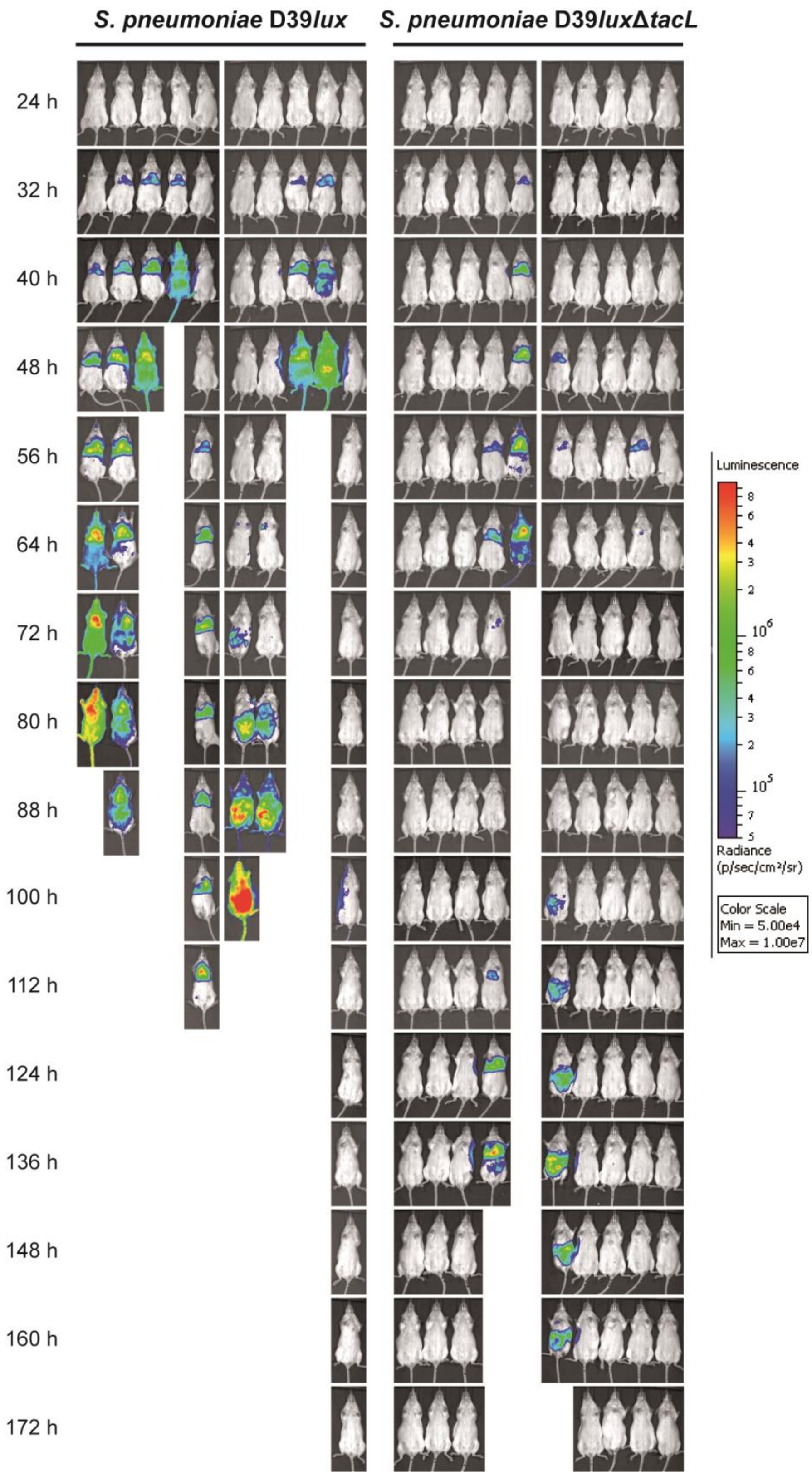

Supplementary Figure 14: Impact of TacL on pneumococcal virulence in the acute pneumonia model. Real-time monitoring of mice (n = 10) intranasally infected with ~2.5 × 10^7 bioluminescent *S. pneumoniae* D39*lux* wild-type or D39*lux*Δ*tacL*. Pneumococcal dissemination was monitored at indicated time points by determination of the luminescence intensity (photons/second) measured with the IVIS® Spectrum system.

Supplementary Table 1. ^{1}H (700.4 MHz), ^{13}C NMR (176.1 MHz), and ^{31}P NMR (283.5 MHz) chemical shift data (δ, ppm) [*J*, Hz] of pnWTA bound to small PGN saccharides from *S. pneumoniae* D39Δ*cps*Δ*lgt*. (respective structure including assignment of residues as well as corresponding ^{1}H, ^{13}C-HSQC-NMR spectrum shown in Supplementary Fig. 2). *non-resolved multiplet; without *P*-Cho: §4.58 [8.5], 101.9; §§5.07 [3.8], *94.1*.

Residue<br>(assignment)	H-1<br>*C-1*	H-2<br>*C-2*	H-3<br>*C-3*	H-4<br>*C-4*	H-5<br>*C-5*	H-6<br>*C-6*	NAc
→3)-α-AATGal*p*-<br>(1→*P* (**A'**)	5.51-5.47*<br>*94.4 [5.5]*	4.23-4.18*<br>*48.6*	4.37-4.32*<br>*75.3*	4.00-3.96*<br>*55.1*	4.53-4.49*<br>*64.4*	1.38-1.33*<br>*16.8*	2.07<br>*22.8*<br>*175.5*
P→6)-β-D-Glc*p*-(1→<br>(**B'**)	4.66-4.62*<br>*104.8*	3.35-3.30*<br>*73.2*	3.50-3.45*<br>*75.7*	3.57-3.51*<br>*69.3*	3.56-3.51*<br>*75.0*	4.16-4.12*<br>*64.9-64.7**	
→1)-ribitol-(5→ *P*<br>(**C**)	3.97-3.93*<br>3.87-3.83*<br>*71.2*	4.02-3.97*<br><br>*71.2*	3.78-3.74*<br><br>*72.0*	3.91-3.87*<br><br>*71.3*	4.07-4.02*<br>4.00-3.95*<br>*67.2 [5.4]*		
→3)-β-D-6-*O*-*P*-Cho-<br>Gal*p*NAc (1→ (**D**)	4.63-4.58*<br>*101.9*	4.13-4.06*<br>*51.2*	3.88-3.83*<br>*75.3*	4.19-4.15*<br>*63.8*	3.85-3.81*<br>*74.1 [8.1]*	4.09-4.03*<br>*65.1 [4.8]*	2.08<br>*23.0*<br>*175.4*
→4)-α-D-6-*O*-*P*-Cho-<br>Gal*p*NAc (1→ (**E**)	5.16 [3.5]<br>*93.9*	4.34-4.30*<br>*49.9*	3.94-3.91*<br>*67.4*	4.11-4.08*<br>*77.2*	4.02-3.98*<br>*71.3*	4.04-3.96*<br>*64.3-64.1**	2.05<br>*22.5*<br>*175.3*
→3)-α-AATGal*p*-(1→<br>(**A**)	4.95 [3.4]<br>*98.8*	4.26-4.20*<br>*48.8*	4.39-4.34*<br>*75.6*	3.95-3.89*<br>*55.2*	4.79-4.73*<br>*63.8*	1.23 [6.4]<br>*16.4*	2.10<br>*22.4*<br>*175.2*
P→6)-β-D-Glc*p*-(1→<br>(**B**)	4.64-4.60*<br>*104.7*	3.35-3.31*<br>*73.4*	3.52-3.47*<br>*76.0*	3.55-3.50*<br>*69.3*	3.59-3.54*<br>*75.0 [7.9]*	4.11-4.02*<br>*64.9-64.7**	
→3)-β-D-6-*O*-*P*-Cho-<br>Gal*p*NAc (1→ (**D**^{term})	4.63-4.58*,§<br>*101.9*	4.13-4.06*<br>*51.2*	3.85-3.81*<br>*75.4*	4.16-4.13*<br>*63.9*	3.85-3.81*<br>*74.1*	4.09-4.03*<br>*65.1 [4.8]*	2.08<br>*23.0*<br>*175.4*
→4)-α-D-6-*O*-*P*-Cho-<br>Gal*p*NAc (**E**^{term})	5.08 [3.8]§§<br><br>*94.2*	4.23-4.20*<br><br>*49.9*	3.82-3.78*<br><br>*68.2*	4.05-4.02*<br><br>*68.6*	4.00-3.96*<br><br>*70.8*	4.08-4.04*<br>4.03-3.98*<br>*65.4 [4.8]*	2.04<br><br>*22.5*<br>*175.3*
Cho-*P*-(6-*O*→<br>@ **D**, **D**^{term}, **E**^{term}	4.35-4.31*<br>*60.1*	3.70-3.66*<br>*66.6*	3.23<br>54.5				
Cho-*P*-(6-*O*→<br>@ **E**	4.30-4.26*<br>*60.0*	3.68-3.65*<br>*66.6*	3.23<br>54.5				
^{31}**P**	*P*-5^{C}/6$^{B/B'}$ 1.91; *P*-6$^{D/D(term)}$/CH_2O^{Cho} 0.32; *P*-6$^{E(term)}$/CH_2O^{Cho} 0.11; *P*-6^{E}/CH_2O^{Cho} −0.16;<br>P-1$^{A'}$/6^{MurNAc} −1.08, −1.16, −1.26.						

Supplementary Table 2. Primer, strains, and antibodies used in this study

Primer	Sequence 5`- 3`	Restriction site
SPD1672_OLup_for	GCGCGC**GCATGC**TTGGAGTAGTAGATGTCAAGGATATCC	*Sph*I
SPD1672_OLdwn_rev	GCGCGC**GAGCTC**GATTTTTTTTCATTTTCTACTCCTCTG	*Sac*I
InvrevKpnISPD1672	GCGCGC**GGTACC**AATGAATCCTTTCTCTCCAAATCTGC	*Kpn*I
InvforPstISPD1672	GCGCGC**CTGCAG**GTTTTATAAGTTTGAAATCTTCTACC	*Pst*I
InvrevKpnIErm	GCGCGC**GGTACC**ACGGTTCGTGTTCGTGCTGACTTGC	*Kpn*I
InforPstIErm	GCGCGC**CTGCAG**GTAGGCGCTAGGGACCTCTTTAGC	*Pst*I
1672_com_for	GCGC**CCATGG**CGAAATCAATAGGCTTTATTG	*Nco*I
Spd1672_com_rev	GCGC**AAGCTT**TTAATCCGTCATGTCCGATAC	*Hind*III
qP_tacL_F	CGAACTGCCTTTCCTGCTAT	
qP_tacL_R	TAAGCCAAAAGGCCTTCCAG	
EnoRT_F	CGGACGTGGTATGGTTCCA	
EnoRT_R	TAGCCAATGATAGCTTCAGCA	

Strains	Resistance	Source of Reference
E2 *E. coli* DH5α	None	Betheseda Research Labs, Gaithersburg, USA
E5 *E. coli* DH5α pUC18	Amp^R	ThermoFisher (#SD0051)
1046 *E. coli* DH5α pUC18Δ*tacL*	Amp^R, Erm^R	This work
1058 *E. coli* DH5α pBAV-*tacL*	Cm^R	This work
SP257 *S. pneumoniae* D39	None	NCTC7466
PN111 *S. pneumoniae* D39Δ*cps*	Km^R	[1]
PN220 *S. pneumoniae* D39Δ*cps*Δ*lgt*	Km^R, Erm^R	[2]
PN602 *S. pneumoniae* D39Δ*tacL*	Erm^R	This work
PN601 *S. pneumoniae* D39Δ*cps*Δ*tacL*	Km^R, Erm^R	This work
PN635 *S. pneumoniae* D39Δ*tacL* pBAV-*tacL*	Erm^R, Cm^R	This work
PN634 *S. pneumoniae* D39Δ*cps*Δ*tacL* pBAV-*tacL*	Km^R, Erm^R, Cm^R	This work
PN149 *S. pneumoniae* D39*lux*	Km^R	[3]
PN642 *S. pneumoniae* D39*lux*Δ*tacL*	Km^R, Erm^R	This work
PN259 *S. pneumoniae* TIGR4Δ*cps*	Km^R	[4]
PN603 *S. pneumoniae* TIGR4Δ*cps*Δ*tacL*	Km^R, Erm^R	This work
PN636 *S. pneumoniae* TIGR4Δ*cps*Δ*tacL* pBAV-*tacL*	Km^R, Erm^R, Cm^R	This work
		This work
**Plasmids**		
pUC18*tacL*	Amp^R, Erm^R	This work
pBAV1C-pE	Cm^R	This work
pBAV-*tacL*	Cm^R	This work
pTP1	Km^R, Erm^R,	[5]

Antibodies	Dilution	Origin	Reference or source
Goat Anti-Mouse IgG H&L (Alexa Fluor® 488)	1:500	Goat	Abcam (#ab150113)
Goat Anti-Rabbit IgG H&L (Alexa Fluor® 488)	1:500	Goat	Abcam (#ab150077)
Goat Anti-Rat IgG H&L (Alexa Fluor® 488)	1:2000	Goat	Abcam (#ab150157)
IRDye® 800CW Goat α-mouse IgG	1:15000	Goat	LI-COR
IRDye® 680RD Goat α-rabbit IgG	1:15000	Goat	LI-COR
Polyclonal PspC-SH13 - IgG	1:500	Mouse	This work
Polyclonal Pce - IgG	1:500	Mouse	This work
Polyclonal CbpL - IgG	1:500	Mouse	[6]
Polyclonal CbpG - IgG	1:500	Rabbit	This work
Polyclonal CbpJ - IgG	1:500	Rabbit	This work
Polyclonal LytA - IgG	1:500	Mouse	This work

Polyclonal PspA-QP2 - IgG	1:500	Mouse	This work
Polyclonal Enolase serum	1:25000	Rabbit	[7]
Kappa murine myeloma clone TEPC 15 – IgA	1:500	Mouse	Sigma (#M1421)
Monoclonal Forssman IgG	1:250	Rat	Provided by J. Müthing, Münster, Germany[2,8]
Polyclonal α-pneu IgG	1:500	Rabbit	Davids Biotechnologie GmbH

Supplementary Table 3. Summary of variants in genome sequence data of *S. pneumoniae* D39Δ*cps*Δ*tacL*.[a]

S. pn. **D39 reference base position**	***S. pn.*** **D39 reference base**	**Type**	***S. pn.*** **D39 Δ*cps*Δ*tacL* Base**	**Predicted effect**	**Gene**	**Function**
423440	A	SNP[b]	G	unknown	intergenic	no feature annotated
780272	A	SNP[b]	G	Asp297Gly	*spd_0768*	AI-2E family transporter
1211772	C	SNP[b]	T	Ala141Thr	*spd_1179*	lanthionine synthetase
1258973	C	SNP[b]	T	unknown	intergenic	no feature annotated

[a]Table shows all variations in genome sequence data of *S. pneumoniae* D39Δ*cps*Δ*tacL* (*S. pn.* D39Δ*cps*Δ*tacL*) in comparison to its parental strain identified by mapping of the sequence reads to the *S. pneumoniae* (*S.pn.*) D39 genome sequence [NC_008533]. Raw sequence data of this study have been submitted to the Short Read Archive (SRA) of the European Nucleotide Archive ENA [RJEB18558].
[b]SNP, single nucleotide polymorphism.

Supplementary Table 4. Summary of variants in genome sequence data of *S. pneumoniae* TIGR4Δ*cps*Δ*tacL*.[a]

S. pn. **TIGR4 reference base position**	***S. pn.*** **TIGR4 reference base**	**Type**	***S. pn*****. TIGR4 Δ*cps*Δ*tacL* Base**	**Predicted effect**	**Gene**	**Function**
1593439	CTTTTTT TTTTTTA	DEL[b]	CTTTTTT TTTTTA	unknown	intergenic	no feature annotated
1798175	G	SNP[c]	T	unknown	Intergenic	no feature annotated
1798221	TAAAAAAT	INS[d]	TAAAAAAAT	unknown	intergenic	no feature annotated
1799483*	A	SNP[c]	T[#]	Leu46Gln	*sp_rs12410*[§]	pseudogene
1799605*	A	SNP[c]	T[#]	Tyr5stop	*sp_rs12410*[§]	pseudogene
1799605*	A	SNP[c]	T[#]	Ser479Thr	*sp_1894*	sucrose phosphorylase
1799744*	T	SNP[c]	C	Glu432Glu	*sp_1894*	sucrose phosphorylase
1799932*	T	SNP[c]	C	Ile370Val	*sp_1894*	sucrose phosphorylase
1826271*	TCC	DEL[b]	TC	Asn22fs[e]	*sp_1914*	membrane protein

[a]Table shows all variations in genome sequence data of *S. pneumoniae* TIGR4Δ*cps*Δ*tacL* (*S. pn.* TIGR4Δ*cps*Δ*tacL*) in comparison to its parental strain identified by mapping of the sequence reads to the *S. pneumoniae* (*S.pn.*) TIGR4 genome sequence [NC_003028.3]. Raw sequence data of this study have been submitted to the Short Read Archive (SRA) of the European Nucleotide Archive ENA [RJEB18558].
[b]DEL, deletion.
[c]SNP, single nucleotide polymorphism.
[d]INS, insertion.
[e]fs, frameshift
*SNPs were verified by PCR amplification of the corresponding gene region, cloning of the PCR product into plasmid pMiniT™ (Promega) followed by DNA sequencing.
[#]SNPs could be excluded by PCR amplification of the corresponding gene region, cloning of the PCR product into plasmid pMiniT™ (Promega) followed by DNA sequencing.
[§]RefSeq annotation NC_003028.3 was generated using the automatic NCBI Prokaryotic Genome Annotation Pipeline 4.1, which indicates *sp_rs12410* as pseudogene that harbors in the published DNA sequence an internal stop codon at position 1799572-1799574.

Supplementary References

1. Rennemeier, C. et al. Thrombospondin-1 promotes cellular adherence of Gram-positive pathogens *via* recognition of peptidoglycan. *Faseb J* **21**, 3118–3132 (2007).
2. Gisch, N. et al. Structural reevaluation of *Streptococcus pneumoniae* lipoteichoic acid and new insights into its immunostimulatory potency. *J Biol Chem* **288**, 15654–15667 (2013).
3. Jensch, I. et al. PavB is a surface-exposed adhesin of *Streptococcus pneumoniae* contributing to nasopharyngeal colonization and airways infections. *Mol Microbiol* **77**, 22–43 (2010).
4. Schulz, C. et al. Regulation of the arginine deiminase system by ArgR2 interferes with arginine metabolism and fitness of *Streptococcus pneumoniae*. *MBio* **5**, e01858-14 (2014).
5. Saleh, M. et al. Molecular architecture of *Streptococcus pneumoniae* surface thioredoxin-fold lipoproteins crucial for extracellular oxidative stress resistance and maintenance of virulence. *EMBO Mol Med* **5**, 1852–1870 (2013).
6. Gutiérrez-Fernández, J. et al. Modular architecture and unique teichoic acid recognition features of choline-binding protein L (CbpL) contributing to pneumococcal pathogenesis. *Sci Rep* **6**, 38094 (2016).
7. Hammerschmidt, S. et al. The host immune regulator factor H interacts via two contact sites with the PspC protein of *Streptococcus pneumoniae* and mediates adhesion to host epithelial cells. *J Immunol* **178**, 5848–5858 (2007).
8. Müthing, J. et al. Promiscuous Shiga toxin 2e and its intimate relationship to Forssman. *Glycobiology* **22**, 849–862 (2012).
